# ACSL4-mediated astrocyte ferroptosis augments neuroinflammation and exacerbates NMOSD pathology

**DOI:** 10.1038/s41418-026-01692-y

**Published:** 2026-03-03

**Authors:** Haixia Wen, Yinyu Zi, Zhuhe Liu, Yunmeng Bai, Jingfang Lin, Haitao Wang, Bingtian Xu, Jigang Wang, Honghao Wang

**Affiliations:** 1 Department of Neurology, Center for Medical Research on Innovation and Translation, Institute of Clinical Medicine, Guangzhou First People’s Hospital, School of Medicine, South China University of Technology, Guangzhou, China; 2 Department of Neurology, Nanfang Hospital, Southern Medical University, Guangzhou, China; 3 Department of Nephrology, Shenzhen Key Laboratory of Kidney Diseases, Shenzhen People’s Hospital, the First Affiliated Hospital, School of Medicine, Southern University of Science and Technology, Shenzhen, China; 4 Guangdong Provincial Key Laboratory of New Drug Screening, School of Pharmaceutical Sciences, Southern Medical University, Guangzhou, China

**Keywords:** Immunological disorders, Inflammation

## Abstract

Neuromyelitis optica spectrum disorder (NMOSD) is recognized as a form of astrocytopathy; however, the mechanisms underlying aquaporin (AQP)4-IgG-induced astrocytic dysfunction remain to be fully elucidated. Here, single-nucleus RNA sequencing revealed that astrocytic ferroptosis is observed in mouse models of NMOSD, accompanied by expression alterations of multiple ferroptosis regulators. The activation of ferroptosis in astrocytes was further confirmed in in vitro NMOSD models through increased intracellular Fe²⁺ levels, lipid peroxidation, malondialdehyde, and lactate dehydrogenase levels, alongside reduced glutathione levels. Moreover, a remarkable increase in inflammatory reactive astrocytes was observed both in vivo and in vitro during NMOSD pathology. Notably, acyl-CoA synthetase long-chain family member 4 (ACSL4) upregulation in astrocytes was validated in NMOSD models. AQP4-IgG-induced ACSL4 upregulation was reversed by early growth response 1 (Egr1) siRNA. Suppressing ACSL4 expression mitigated astrocytic ferroptosis, reduced reactive astrocytes, attenuated demyelination, and ultimately improved NMOSD prognosis in mice. These findings demonstrate that ACSL4 mediates astrocytic ferroptosis, thereby contributing to NMOSD progression. Targeting ACSL4 may represent a promising astrocyte-directed therapeutic strategy for NMOSD.

## Introduction

Neuromyelitis optica spectrum disorder (NMOSD) is a chronic neuroinflammatory autoimmune disease that primarily affects the optic nerve and spinal cord [1, 2]. More than 80% of NMOSD patients are seropositive for aquaporin-4-immunoglobulin G (AQP4-IgG), the pathogenic autoantibody targeting aquaporin-4 (AQP4), a water channel protein predominantly expressed on astrocyte end-feet [2]. The binding of AQP4-IgG to AQP4 initiates the formation of the membrane attack complex, leading to astrocytic damage, which in turn causes secondary oligodendrocyte and neuron injury [2–4]. Consequently, NMOSD pathology is recognized as a form of astrocytopathy. However, the precise mechanisms underlying AQP4-IgG-induced astrocytic dysfunction remain to be fully elucidated. Emerging evidence suggests that astrocytes are not merely passive targets of immune-mediated damage but actively contribute to the inflammatory cascade that underlies NMOSD progression [5–7]. Thus, understanding the interplay between AQP4-IgG binding and astrocytic responses is crucial for identifying novel therapeutic targets.

AQP4-IgG binding to AQP4 primarily induces astrocytic injury through activation of the classical complement cascade [8–10]. Additionally, this interaction triggers a cascade of responses that promote a reactive and inflammatory astrocytic phenotype, which recruits innate immune cells into the CNS. This further amplifies tissue dyshomeostasis and exacerbates injury in NMOSD [11]. Although significant progress has been made in understanding the temporal aspects of astrocytopathy in NMOSD [3, 5], the pathophysiological mechanisms governing the proinflammatory modulation of reactive astrocytes remain largely unknown. Identifying upstream regulators of proinflammatory astrocyte activation may provide insight into how NMOSD-associated astrocytopathy.

Ferroptosis is an iron-dependent programmed cell death pathway, characterized by excessive lipid peroxidation and iron-dependent reactive oxygen species production [12, 13]. Several key regulators of ferroptosis have been identified, including acyl-CoA synthetase long-chain family member 4 (ACSL4), solute carrier family 7 member 11 (SLC7A11), nuclear receptor coactivator 4 (NCOA4), cyclooxygenase 2 (COX2), ferritin heavy chain 1 (FTH1), glutathione peroxidase 4 (GPX4), and glutathione (GSH) [14–16]. Unlike apoptosis and necrosis, ferroptosis is uniquely driven by iron-catalyzed lipid peroxidation [17], rendering it particularly relevant in CNS disorders where iron homeostasis is disrupted. Accumulating evidence suggests that iron overload and ferroptosis play crucial roles in the pathogenesis of demyelinating diseases such as multiple sclerosis and neuromyelitis optica [18–20]. Notably, the role of astrocytic ferroptosis in CNS disorders has garnered increasing attention [21–23]. The vulnerability of astrocytes to ferroptotic stress suggests that targeting ferroptosis-related pathways may hold promise for preserving astrocyte function and limiting secondary neuronal damage in NMOSD.

To date, the precise involvement of astrocytic ferroptosis in NMOSD and its molecular regulatory mechanisms remains unknown. Our research has yet to delineate whether ferroptosis serves as a primary driver of astrocyte dysfunction in NMOSD. By investigating the upstream triggers and downstream effects of astrocytic ferroptosis, our research clarifies the contribution of astrocytic ferroptosis to NMOSD pathology and reveals novel intervention points.

## Results

### Astrocytic ferroptosis is triggered in the NMOSD model in vivo and in vitro

**Fig. 1:**
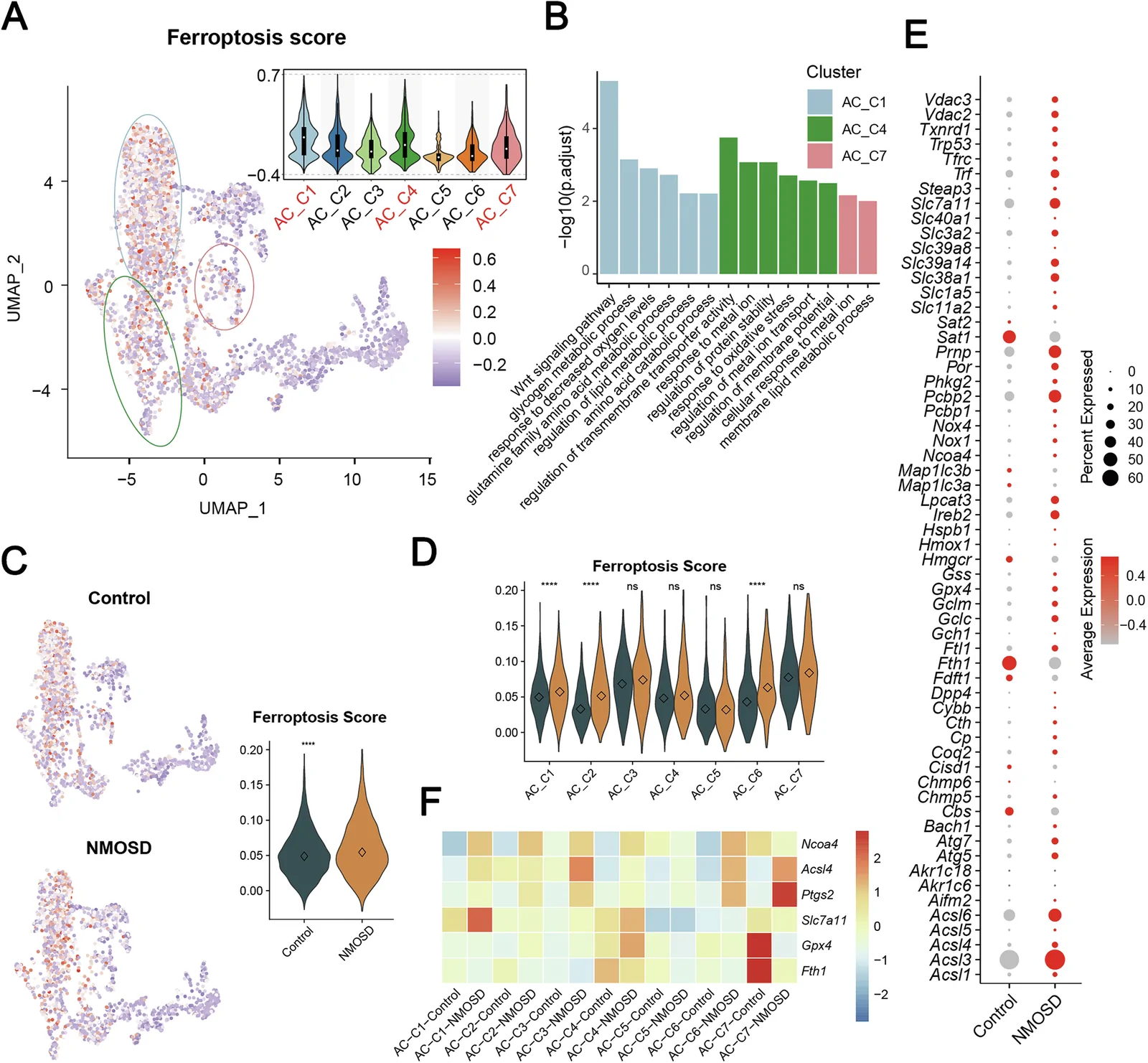
Transcriptional characteristics of astrocytes from NMOSD models in vivo. Passive immunization NMOSD mice model was established via intrathecal injection of with patient-derived AQP4-IgG to collect striatum tissue from the lesion site for snRNA-seq. **A** Volcano plot showing ferroptosis score in astrocytes from Control mice. **B** KEGG barplot showing the level of different ferroptosis-related metabolic pathways. **C** Violin plot showing ferroptosis score between NMOSD mice vs Control mice using wilxoc test. **D** Violin plot showing ferroptosis score in each tissue between NMOSD vs Control group using the Wilcox test. **E** Dot plot showing the expression levels of representative genes for each group. **F** Heatmap showing the expression of ferropotosis-related DEGs in different groups. ns not significant; **** *p* < 0.0001.

Passive immunization NMOSD mice model was established to delve deeper into the variance of astrocytes during NMOSD pathology. Striatum tissue from the lesion site of NMOSD mice was collected for snRNA-seq. According to our recent report, after data quality control at both gene and cell levels, differential gene expression analysis contrasting NMOSD mice with the Control group identified 4381 differentially expressed genes (DEGs) in astrocytes, including 4324 upregulated genes and 57 downregulated genes [8]. DEGs pathway analysis based on Gene Ontology and Kyoto Encyclopedia of Genes and Genomes (KEGG) indicated the involvement of multiple ferroptosis-related metabolic pathways (Fig. 1A, B), such as lipid metabolic pathway, oxidative stress, and metal ion. Notably, ferroptosis activation in astrocytes from NMOSD mice was confirmed via ferroptosis score analysis (Fig. 1C, D). Furthermore, expression profiles of ferroptosis-related modulators in astrocytes revealed distinct differences (Fig. 1E). These alterations suggest a fundamental shift in the metabolic and oxidative state of astrocytes during NMOSD, highlighting a potential ferroptosis-mediated mechanism that exacerbates neuroinflammation. The upregulation of ACSL4, a key enzyme in lipid remodeling, aligns with previous findings linking lipid peroxidation to ferroptotic vulnerability. Conversely, the dysregulation of GPX4, a major ferroptosis suppressor, may indicate an impaired antioxidant defense, rendering astrocytes more susceptible to oxidative damage. Among these modulators, we specifically assessed the expression of key ferroptosis regulators and markers, including ACSL4, GPX4, FTH1, COX2 (PTGS2), NCOA4, and SLC7A11. As shown in Fig. 1F, the expression levels of ACSL4, NCOA4, and COX2 (PTGS2) exhibited an upward trend, whereas FTH1 expression was downregulated in NMOSD mice compared to Control mice. Additionally, SLC7A11 and GPX4 expression levels displayed inconsistent changes (Fig. 1F).

**Fig. 2:**
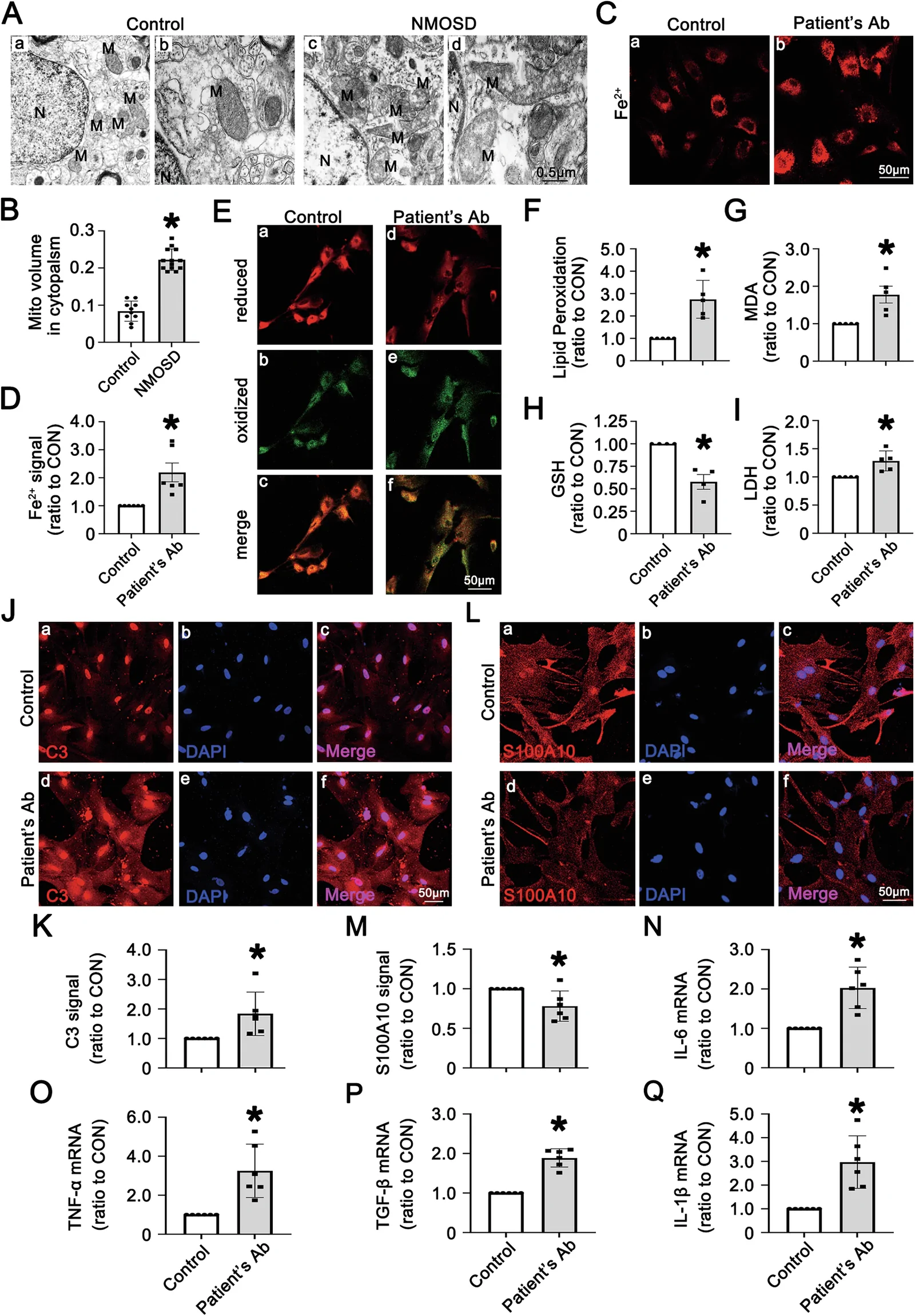
Astrocytic ferroptosis and proinflammatory activity were activated in the NMOSD model. Spinal cord tissues from passive immunization NMOSD and Control mice were analyzed with TEM. **A** Representative TEM micrograph showing mitochondria ultrastructure, mitochondrial morphology from spinal cord with patient-derived AQP4-IgG treatment (N: nucleus; M: mitochondria). **B** Histogram showing the cytoplasmic volume fractions of mitochondria in (**A**) (*n* ≥ 10). Primary astrocytes were treated with AQP4-IgG or CON-IgG for 12 h to induce an in vitro NMOSD model. Intracellular Fe²⁺ levels in primary astrocytes were measured using FerroOrange. **C** Representative fluorescence images showing intracellular Fe^2+^ signal in primary astrocytes with AQP4-IgG treatment. **D** Histogram showing astrocytic Fe^2+^ content in (**C**) (*n* = 6). Intracellular lipid peroxidation was assessed using the Image-iT^®^ Lipid Peroxidation Kit. **E** Representative fluorescence images showing the reduced and oxidized signals of lipid peroxidation dye in primary astrocytes with AQP4-IgG treatment. **F** Histogram showing the level of lipid peroxidation in (**E**) (*n* = 5). **G** Histogram showing MDA level in primary astrocytes (*n* = 5). **H** Histogram showing GSH level in primary astrocytes (*n* = 4). **I** Histogram showing LDH level in primary astrocytes (*n* = 5). **J** Representative fluorescence images showing intracellular C3 signal in primary astrocytes with or without patient-derived AQP4-IgG treatment. **K** Histogram showing C3 expression in primary astrocytes (*n* = 6). **L** Representative fluorescence images showing S100A10 signal in primary astrocytes with or without patient-derived AQP4-IgG treatment. **M** Histogram showing S100A10 expression in primary astrocytes (*n* = 6). **N** Histogram showing IL-6 RNA level in primary astrocytes (*n* = 6). **O** Histogram showing TNF-α RNA level in primary astrocytes (*n* = 6). **P** Histogram showing TGF-β RNA level in primary astrocytes (*n* = 6). **Q** Histogram showing IL-1β RNA level in primary astrocytes (*n* = 6). Data are expressed as a ratio of the value of the Control group. Each bar represents the mean ± SEM. *p < 0.05 versus the control group.

To further confirm the role of ferroptosis in astrocytic inflammation during NMOSD pathology, ferroptosis markers were examined. Mechanistically, disordered mitochondrial homeostasis has been highlighted as an important factor to induce ferroptosis [24, 25]. Representative transmission electron microscopy images revealed that the NMOSD group displayed abnormal mitochondrial morphology in the spinal cord, including cristae rupture and increased membrane density (Fig. 2A, B). Given that iron accumulation and uncontrolled lipid peroxidation are hallmarks of ferroptosis [14, 26], iron content and lipid peroxidation levels were assessed in the Control and NMOSD groups. As shown in Fig. 2, astrocytic Fe²⁺ content was significantly elevated following patient-derived AQP4-IgG stimulation, compared to the Control group (Fig. 2C, D). Similarly, lipid peroxidation levels were increased in primary astrocytes upon AQP4-IgG treatment, compared to the Control group (Fig. 2E, F). AQP4-IgG treatment also elevated cellular levels of MDA and LDH while reducing GSH levels in primary astrocytes (Fig. 2G–I).

We further found that treatment with ferroptosis inhibitor Liproxstatin-1 (Lip-1, 100 nM) suppressed the increase in astrocyte death caused by AQP4-IgG stimulation (Supplementary Fig. 1), which confirms the involvement of ferroptosis in astrocyte damage. Meanwhile, we treated astrocytes with the classical ferroptosis inducers Erastin (40 μM) and RSL3 (0.5 μM). As expected, both compounds potently induced astrocyte death (Supplementary Fig. 2). Furthermore, we found that both Erastin and RSL3 treatment led to a significant downregulation of AQP4 protein levels (Supplementary Fig. 2). This finding suggests the execution of ferroptosis can directly impact the primary target of NMOSD, the AQP4 protein itself.

Given that ferroptosis is a non-apoptotic form of cell death, its involvement in NMOSD pathology may explain the persistence of neuroinflammatory responses even in the absence of overt cell loss. Fluorescent immunohistochemistry demonstrated that AQP4-IgG stimulation promoted C3 expression (Fig. 2J, K), an A1-like proinflammatory reactive astrocyte marker, while inhibiting S100A10 expression (Fig. 2L, M), an A2-like anti-inflammatory astrocyte marker, indicating induction of proinflammatory reactive astrocytes during NMOSD pathology. Furthermore, expression levels of proinflammatory cytokines, including IL-6 (Fig. 2N), TNF-α (Fig. 2O), TGF-β (Fig. 2P), and IL-1β (Fig. 2Q), were significantly upregulated in primary astrocytes following AQP4-IgG stimulation. In conclusion, our discovery indicates that astrocytic ferroptosis is triggered in the NMOSD model.

### ACSL4 is upregulated in astrocytes during NMOSD pathology in vivo and in vitro

**Fig. 3:**
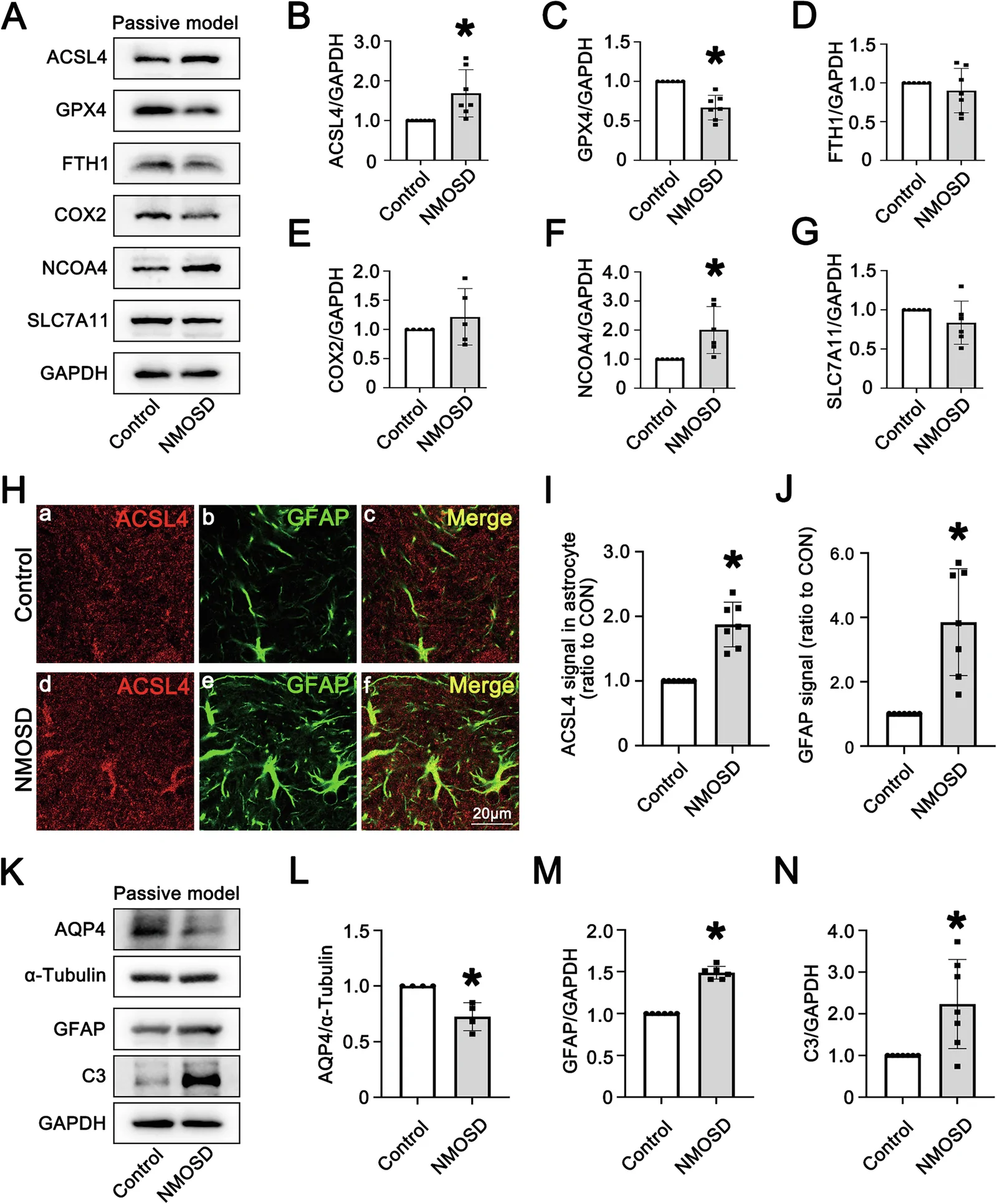
The expressions of ferroptosis-related molecules and the proinflammatory activity in passive immunization of NMOSD mice. Passive immunization NMOSD mice model was established via intrathecal injection with patient-derived AQP4-IgG. On the 5th day after injection, spinal cord tissue from the lesion site was collected for protein detection. **A** Representative immunoblots showing the expressions of ACSL4, GPX4, FTH1, COX2, NCOA4, and SLC7A11 in the spinal cord of passive immunization NMOSD mice. **B** Histogram showing ACSL4 expression in the spinal cord (*n* = 7 mice per group). **C** Histogram showing GPX4 expression in the spinal cord (*n* = 7 mice per group). **D** Histogram showing FTH1 expression in the spinal cord (*n* = 7 mice per group). **E** Histogram showing COX2 expression in the spinal cord (*n* = 5 mice per group). **F** Histogram showing NCOA4 expression in the spinal cord (*n* = 6 mice per group). **G** Histogram showing SLC7A11 expression in the spinal cord (*n* = 6 mice per group). **H** Representative photomicrographs with fluorescent staining of ACSL4 (red) and GFAP (green) in the spinal cord. Scale bar: 20 μm. **I** Histogram showing the fluorescence intensity of ACSL4 signal, which colocalized with GFAP (*n* = 7 mice per group). **J** Histogram showing the fluorescence intensity of GFAP in the spinal cord (*n* = 7 mice per group). **K** Representative immunoblots showing the expressions of AQP4, GFAP, and C3 in the spinal cord of passive immunization NMOSD mice. **L** Histogram showing AQP4 expression in spinal cord (*n* = 4 mice per group). **M** Histogram showing GFAP expression in the spinal cord (*n* = 6 mice per group). **N** Histogram showing C3 expression in the spinal cord (*n* = 7 mice per group). Data are expressed as a ratio of the value of the control mice. Each bar represents the mean ± SEM. *p < 0.05 versus Control mice.

Based on single-nucleus RNA sequencing results, the expression of ferroptosis-related molecules was further validated in vivo and in vitro. Consistent with the single-nucleus RNA sequencing data, western blot analysis confirmed a significant increase in ACSL4 and NCOA4 expression in passive immunization NMOSD mice, accompanied by a decline in GPX4 levels (Fig. 3A). However, no significant differences were observed in the levels of COX2, FTH1, or SLC7A11 between Control and passive immunization NMOSD mice (Fig. 3A–G). The selective upregulation of ACSL4 and NCOA4, alongside GPX4 downregulation, suggests a targeted ferroptotic shift in astrocytes rather than a broad, non-specific oxidative response. The stability of FTH1 and COX2 levels further indicates that NMOSD-associated astrocytopathy may involve specific regulatory nodes within the ferroptosis pathway, rather than generalized metabolic disruption.

Double immunofluorescence staining further demonstrated that astrocytic ACSL4 expression was significantly upregulated in the spinal cord of NMOSD mice compared to Control mice (Fig. 3H a–c, I), as evidenced by increased colocalization of ACSL4 with GFAP (Fig. 3H d–f, I). Additionally, fluorescent GFAP signals were markedly elevated in NMOSD mice compared to Control mice (Fig. 3H, J). AQP4-IgG treatment led to a reduction in AQP4 expression, while C3 and GFAP levels were also upregulated in NMOSD mice, as confirmed by western blot analysis (Fig. 3K–N), further supporting the activation of astrocytic inflammation in vivo.

**Fig. 4:**
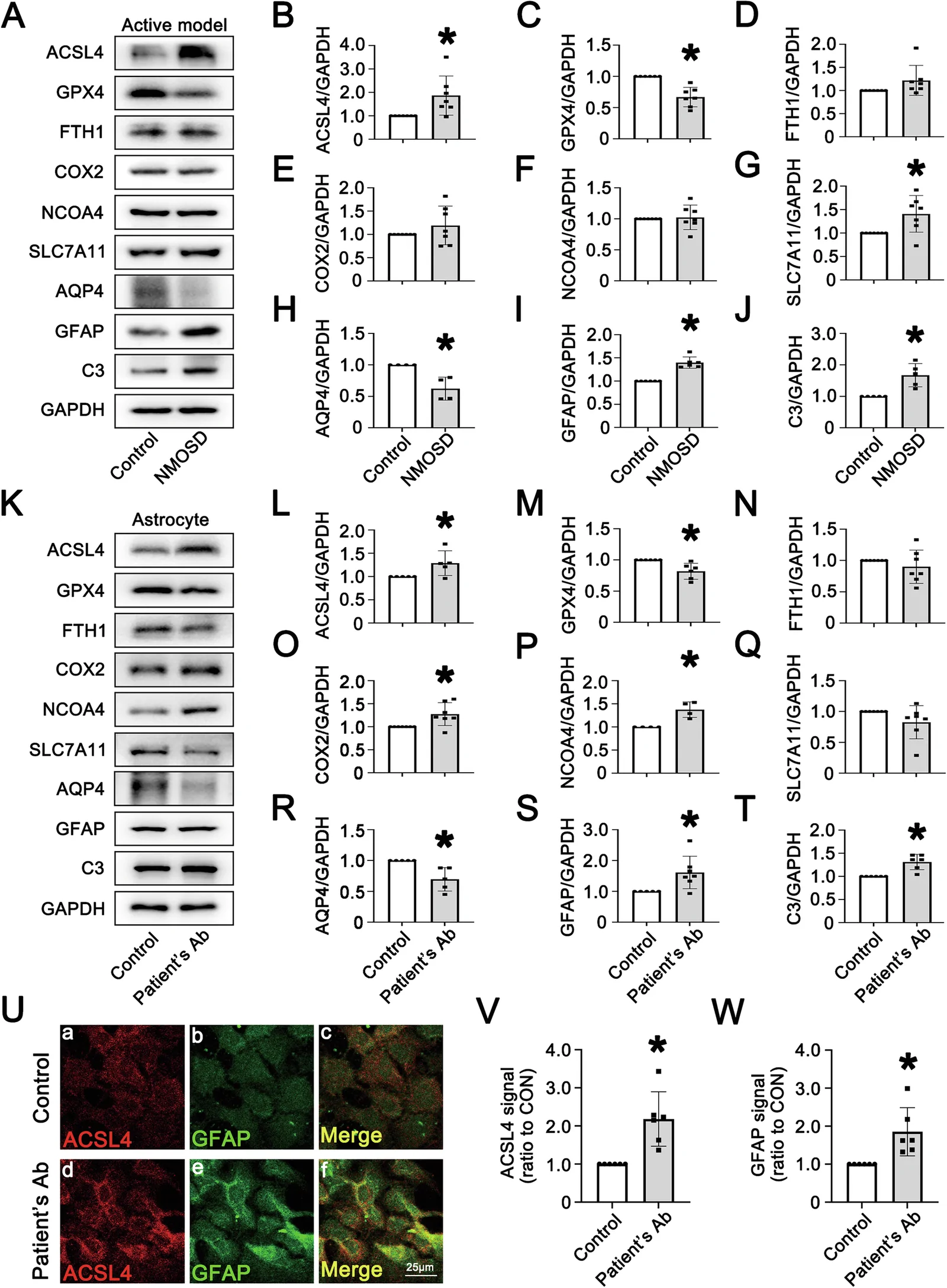
The expressions of ferroptosis-related molecules and the proinflammatory activity of astrocytes in active immunization NMOSD mice and in vitro NMOSD model. Active immunization NMOSD mice model was established via subcutaneous injection of AQP4 p201-220 peptides. At 20th day after injection, spinal cord tissue was collected from the lesion site for protein detection. **A** Representative immunoblots showing the expressions of ACSL4, GPX4, FTH1, COX2, NCOA4, and SLC7A11 in the spinal cord of active immunization NMOSD mice. **B** Histogram showing ACSL4 expression in the spinal cord (*n* = 7 mice per group). **C** Histogram showing GPX4 expression in the spinal cord (*n* = 7 mice per group). **D** Histogram showing FTH1 expression in the spinal cord (*n* = 7 mice per group). **E** Histogram showing COX2 expression in the spinal cord (*n* = 7 mice per group). **F** Histogram showing NCOA4 expression in the spinal cord (*n* = 7 mice per group). **G** Histogram showing SLC7A11 expression in the spinal cord (*n* = 7 mice per group). **H** Histogram showing AQP4 expression in the spinal cord (*n* = 4 mice per group). **I** Histogram showing GFAP expression in the spinal cord (*n* = 6 mice per group). **J** Histogram showing C3 expression in spinal cord (*n* = 5 mice per group). Primary astrocytes were treated with AQP4-IgG or CON-IgG for 12 h to induce an in vitro NMOSD model for protein detection. **K** Representative immunoblots showing the expressions of ACSL4, GPX4, FTH1, COX2, NCOA4, SLC7A11, AQP4, GFAP, and C3 in primary astrocytes exposed to purified AQP4-IgG and hC. **L** Histogram showing ACSL4 expression in primary astrocytes (*n* = 5). **M** Histogram showing GPX4 expression in primary astrocytes (*n* = 6). **N** Histogram showing FTH1 expression in primary astrocytes (*n* = 7). **O** Histogram showing COX2 expression in primary astrocytes (*n* = 7). **P** Histogram showing NCOA4 expression in primary astrocytes (*n* = 4). **Q** Histogram showing SLC7A11 expression in primary astrocytes (*n* = 7). **R** Histogram showing AQP4 expression in primary astrocytes (*n* = 5). **S** Histogram showing GFAP expression in primary astrocytes (*n* = 7). **T** Histogram showing C3 expression in primary astrocytes (*n* = 6). **U** Representative photomicrographs with fluorescent staining of ACSL4 (red) and GFAP (green) in primary astrocytes. Scale bar: 25 μm. **V** Histogram showing the fluorescence intensity of ACSL4 signal (*n* = 6). **W** Histogram showing the fluorescence intensity of GFAP (*n* = 6). Data are expressed as a ratio of the value of the control mice. Each bar represents the mean ± SEM. *p < 0.05 versus Control mice.

Next, a similar upregulation of ACSL4 (Fig. 4A, B) and a decline in GPX4 (Fig. 4A, C) were observed in active immunization NMOSD mice. However, FTH1, COX2, and NCOA4 levels remained unchanged between Control and NMOSD mice (Fig. 4D–F), while SLC7A11 expression was increased in active immunization NMOSD mice (Fig. 4G). Additionally, AQP4 expression was reduced, while C3 and GFAP levels were elevated in active immunization NMOSD mice (Fig. 4H–J). The expression pattern of ferroptosis regulators and astrocyte markers in primary astrocytes was consistent with that observed in passive immunization NMOSD mice, with the exception of a COX2 upregulation induced by AQP4-IgG (Fig. 4K–T). Notably, in agreement with in vivo NMOSD models, patient-derived AQP4-IgG triggered an increase in ACSL4 and GFAP expression in primary astrocytes (Fig. 4U–W).

To further confirm that these ferroptosis-related molecules render the astrocytes potentially sensitive to ferroptosis in NMOSD, we performed genetic knockdown of two key ferroptotic regulatory markers, ACSL4 and GPX4. As shown in Supplementary Fig. 3, knocking down ACSL4 significantly alleviated AQP4-IgG-induced astrocyte death. Conversely, knocking down GPX4 markedly exacerbated astrocyte death upon AQP4-IgG stimulation (Supplementary Fig. 4).

These findings reinforce the notion that ACSL4 is a key driver of NMOSD-related astrocytic dysfunction, potentially serving as a molecular switch between ferroptotic vulnerability and neuroinflammatory amplification.

### ACSL4 upregulation in astrocytes during NMOSD pathology is regulated by Egr1

**Fig. 5:**
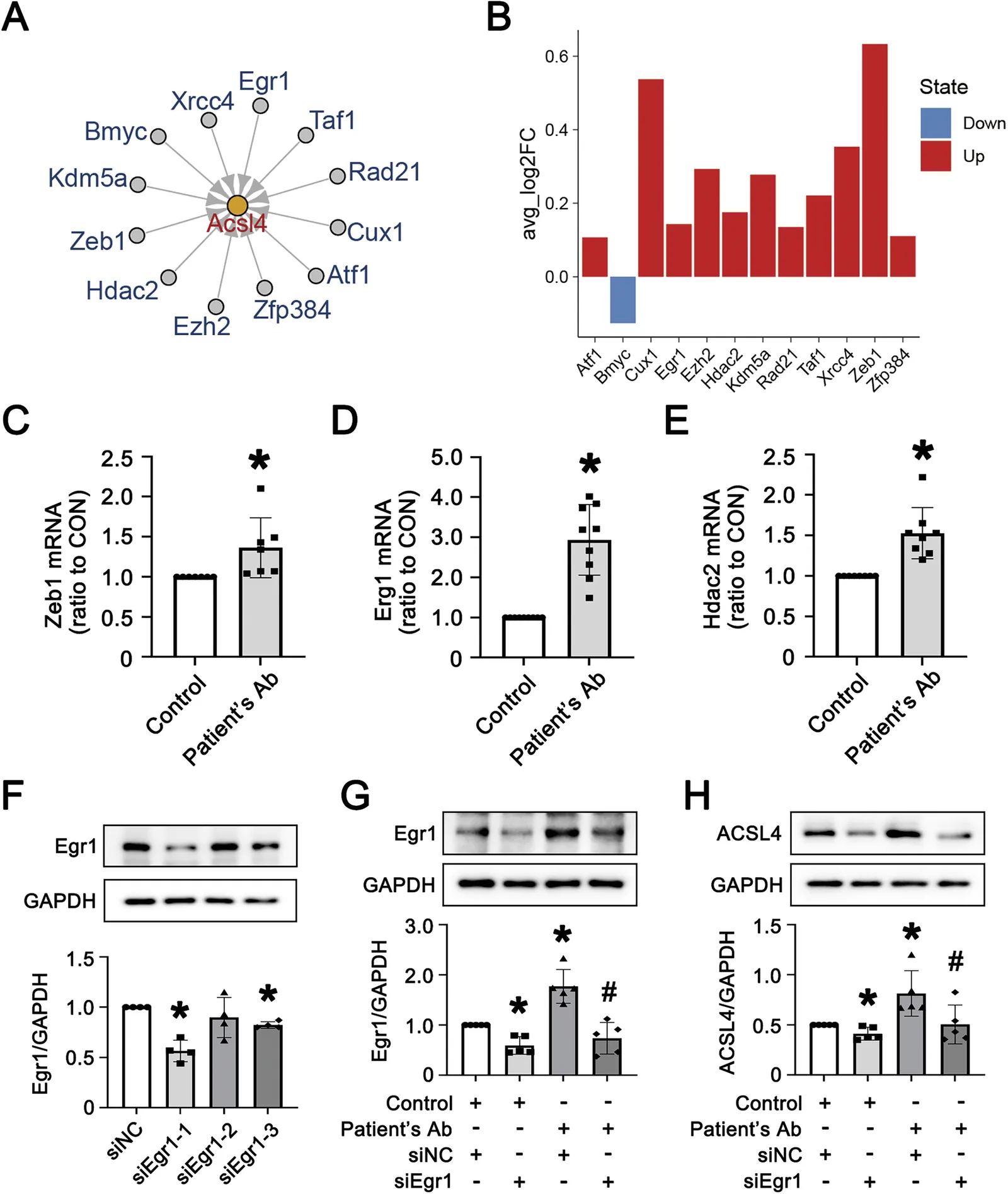
ACSL4 upregulation in astrocytes during NMOSD pathology was under the regulation of Egr1. SCENIC analysis was performed using the up- and down-regulated DEGs identified via snRNA-seq between the NMOSD and Control groups. **A** The regulatory network of ACSL4 and target genes. **B** Bar plot showing the up-and down-regulated level of transcription factors regulating ACSL4. Primary astrocytes were treated with AQP4-IgG or CON-IgG for 12 h. **C** Histogram showing Zeb1 mRNA level in primary astrocytes (*n* = 7). **D** Histogram showing Egr1 mRNA level in primary astrocytes (*n* = 9). **E** Histogram showing Hdac2 mRNA level in primary astrocytes (*n* = 8). **F** Representative immunoblots showing Egr1 expressions in primary astrocytes treated with siNC or Egr1-targeted siRNAs for 24 h (*n* = 4). **G** Representative immunoblots showing Egr1 expression in primary astrocytes transfected with negative control siRNA (siNC) or si-Egr1 followed by treating with AQP4-IgG for 12 h (*n* = 5). **H** Representative immunoblots showing ACSL4 expression in primary astrocytes transfected with siNC or si-Egr1, followed by treatment with AQP4-IgG for 12 h (*n* = 5). Each bar represents the mean ± SEM. *p < 0.05 versus control group treated with siNC. #*p* < 0.05 versus the NMOSD group treated with siNC.

To further investigate the potential upstream regulatory mechanism of ACSL4 upregulation in astrocytes, Single-Cell Regulatory Network Inference and Clustering (SCENIC) analysis was performed using the up- and down-regulated DEGs between the NMOSD and Control groups. As shown in Fig. 5A, 12 potential transcription factors (TFs) were identified, which were mostly enriched in the NMOSD group. Consistent with ACSL4, most of these factors exhibited up-state in NMOSD mice, except for Bmyc (Fig. 5B). Next, the expressions of zinc-finger E-box-binding homeobox 1 (Zeb1, Fig. 5C), early growth response 1 (Egr1, Fig. 5D), and Hdac2 (Fig. 5E), which had been reported to regulate ferroptosis, were confirmed to be enhanced with AQP4-IgG stimulation. The transfection efficiency of Egr1 siRNA was confirmed via western blot analysis. As shown in Fig. 5F, the most effective silencing was achieved using si-Egr1-1, which was selected for subsequent experiments. Furthermore, si-Egr1 effectively suppressed astrocytic Egr1 expression with Con-IgG or AQP4-IgG treatment (Fig. 5G) compared to si-NC. Specifically, the increase of ACSL4 triggered by AQP4-IgG was effectively counteracted by Egr1 silencing in primary astrocytes (Fig. 5H). These findings indicate that Egr1 might promote ACSL4 upregulation in astrocytes during NMOSD pathology.

### ACSL4 silencing suppresses astrocytic ferroptosis and inflammation in the NMOSD model in vitro

**Fig. 6:**
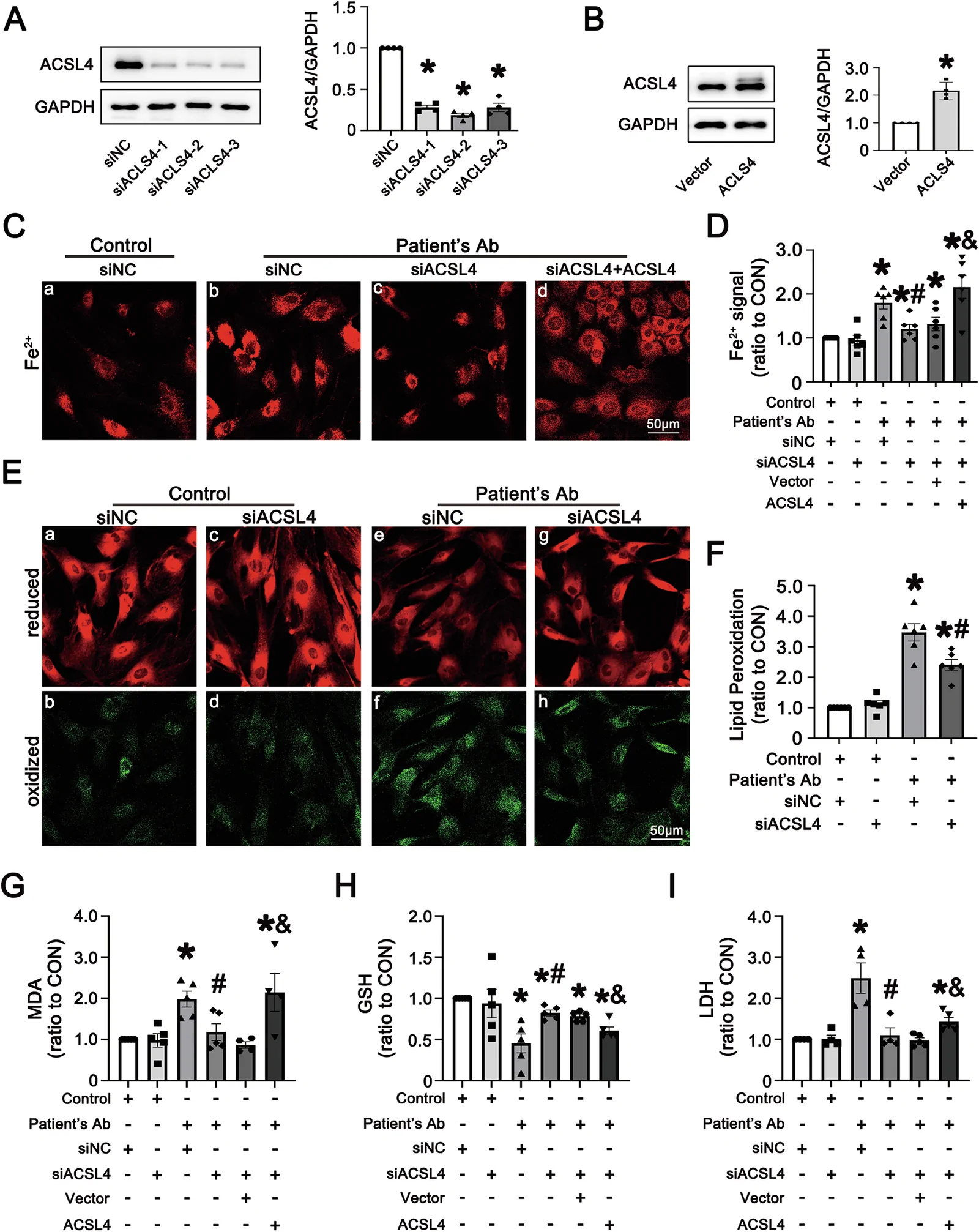
Astrocytic ACSL4 silencing reversed the activation of ferroptosis in the NMOSD model in vitro. Intracellular Fe²⁺ levels in primary astrocytes treated with AQP4-IgG for 12 h were measured using FerroOrange. **A** Representative immunoblots showing ACSL4 expression in primary astrocytes treated with siNC or three ACSL4-targeted siRNA (*n* = 4). Each bar represents the mean ± SEM. *p < 0.05 versus the control group treated with siNC. **B** Representative immunoblots showing ACSL4 expression in primary astrocytes treated with Vector or ACSL4 plasmid (*n* = 4). Data are expressed as a ratio of the value of the Vector + Control group. Each bar represents the mean ± SEM. *p <   0.05 versus control group treated with Vector. **C** Representative fluorescence image showing intracellular Fe^2+^ signal in primary astrocytes. **D** Histogram showing astrocytic Fe^2+^ content (*n* = 6). Intracellular lipid peroxidation was assessed using the Image-iT^®^ Lipid Peroxidation Kit. **E** Representative fluorescence images showing the reduced and oxidized signals of lipid peroxidation dye in primary astrocytes treated with AQP4-IgG for 12 h. **F** Histogram showing the level of lipid peroxidation in primary astrocytes (*n* = 6). **G** Histogram showing MDA level in primary astrocytes treated with AQP4-IgG for 12 h (*n* = 5). **H** Histogram showing GSH level in primary astrocytes treated with AQP4-IgG for 12 h (*n* = 5). **I** Histogram showing LDH level in primary astrocytes treated with AQP4-IgG for 24 h (*n* = 4). Data are expressed as a ratio of the value of siNC + the Control group. Each bar represents the mean ± SEM. *p < 0.05 versus siNC + Control group. #*p* < 0.05 versus siNC + NMOSD group. &*p* < 0.05 versus siNC + NMOSD group treated with Vector.

Given the consistent upregulation of ACSL4 in both in vivo and in vitro NMOSD models, the role of ACSL4 in astrocytic ferroptosis was further explored through ACSL4 silencing and overexpression during NMOSD pathology. The transfection efficiency of ACSL4 siRNA and plasmid was confirmed via western blot analysis (Fig. 6A, B). As shown in Fig. 6A, the most effective silencing was achieved using si-ACSL4-2, which was selected for subsequent experiments.

Compared to negative control siRNA (siNC) group, ACSL4 silencing via ACSL4 siRNA significantly reduced Fe²⁺ accumulation in astrocytes following AQP4-IgG treatment (Fig. 6C, D), without affecting Fe²⁺ levels in Control astrocytes (Fig. 6C, a). This reduction was reversed by ACSL4 overexpression (Fig. 6C, c, d). Additionally, si-ACSL4 treatment suppressed the increase in lipid peroxidation (Fig. 6E, F), MDA, and LDH, while also preventing the AQP4-IgG-induced decline in GSH levels (Fig. 6G–I) in primary astrocytes. However, these effects were reversed upon ACSL4 overexpression, as demonstrated by the restoration of MDA, LDH, and GSH levels (Fig. 6G–I). Meanwhile, si-Egr1 treatment also causes similar effects of si-ACSL4, as evidenced by an increase in GSH and a decrease in lipid peroxidation and MDA caused by AQP4-IgG stimulation (Supplementary Fig. 5).

**Fig. 7:**
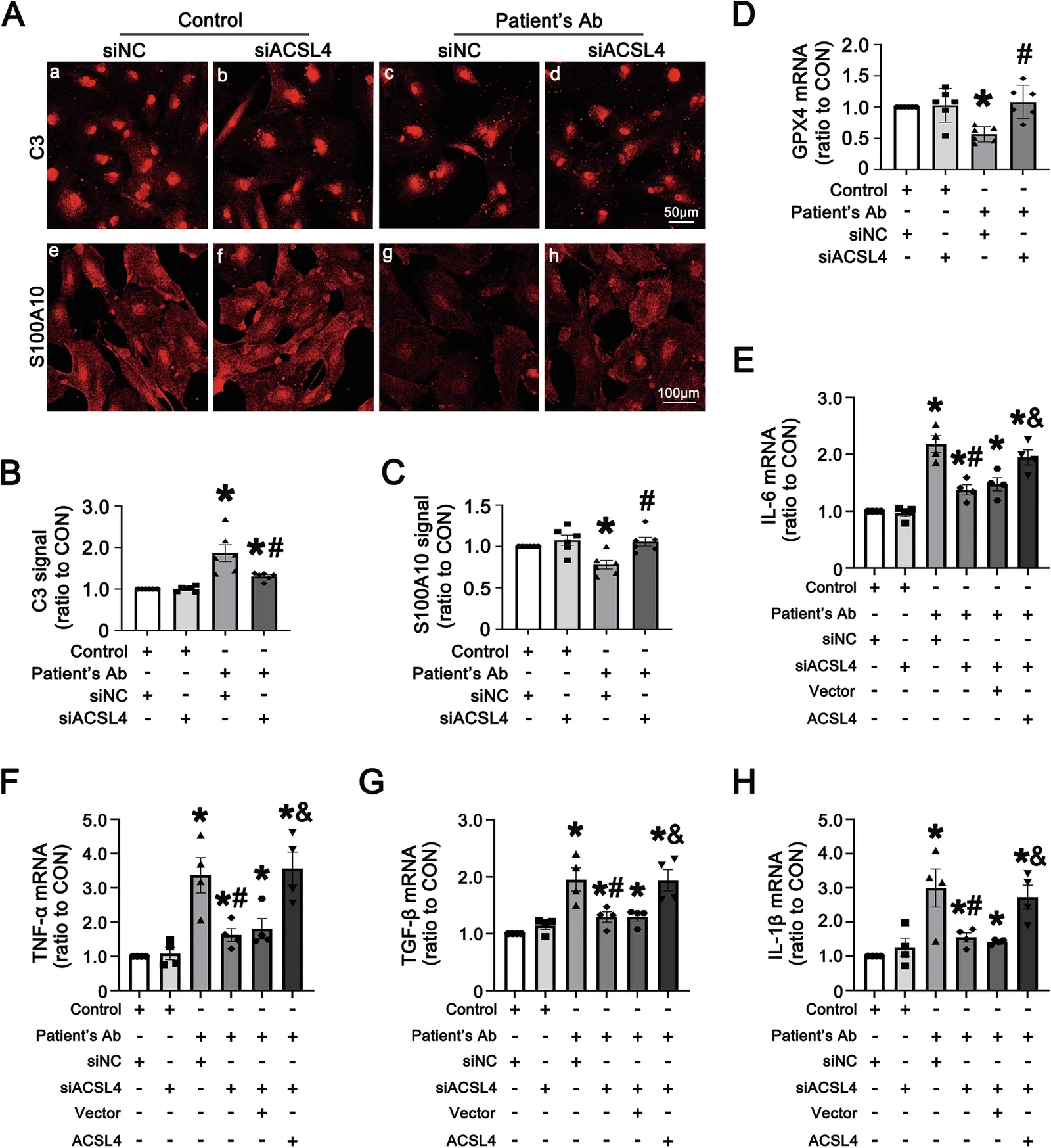
ACSL4 silencing suppressed the activation of astrocytic proinflammatory activity in the NMOSD model in vitro. Primary astrocytes were treated with AQP4-IgG or CON-IgG for 12 h after incubation with siNC or siACSL4. **A** Representative fluorescence images showing intracellular C3 and S100A10 signal in primary astrocytes. Scale bar: a-d, 50 μm; e-h, 100 μm. **B** Histogram showing C3 expression in primary astrocytes (*n* = 6). **C** Histogram showing S100A10 expression in primary astrocytes (*n* = 6). **D** Histogram showing GPX4 RNA level in primary astrocytes (*n* = 6). Primary astrocytes were treated with AQP4-IgG or CON-IgG for 12 h after incubation with siNC or siACSL4. Primary astrocytes were treated with AQP4-IgG or CON-IgG for 12 h after incubation with siNC or siACSL4 and ACSL4 plasmid. **E** Histogram showing IL-6 RNA level in primary astrocytes (*n* = 4). **F** Histogram showing TNF-α RNA level in primary astrocytes (*n* = 4). **G** Histogram showing TGF-β RNA level in primary astrocytes (*n* = 4). **H** Histogram showing IL-1β RNA level in primary astrocytes (*n* = 4). Data are expressed as a ratio of the value of siNC + the Control group. Each bar represents the mean ± SEM. *p < 0.05 versus siNC + Control group. #*p* < 0.05 versus siNC + NMOSD group. & *p* < 0.05 versus siNC+NMOSD group treated with Vector.

Next, the role of ACSL4 in astrocytic inflammation was further examined. As expected, ACSL4 silencing following AQP4-IgG stimulation led to reduced C3 expression (Fig. 7A, a–d) and an increase in S100A10 signal (Fig. 7A, e–h), suggesting an inhibition of proinflammatory reactive astrocyte activation (Fig. 7A–C). Furthermore, GPX4 mRNA level (Fig. 7D) suppressed by AQP4-IgG stimulation was reversed via ACSL4 silencing. Conversely, the elevated mRNA expression levels of IL-6 (Fig. 7E), TNF-α (Fig. 7F), TGF-β (Fig. 7G), and IL-1β (Fig. 7H) induced by AQP4-IgG stimulation were partially suppressed by ACSL4 silencing. Notably, these suppressive effects were reversed upon ACSL4 overexpression via ACSL4 plasmid transfection (Fig. 7E–H).

Taken together, our results demonstrate that ACSL4 silencing suppresses ferroptosis and inflammatory response in astrocytes.

### Astrocytic ACSL4 silencing improves the prognosis of NMOSD mice

**Fig. 8:**
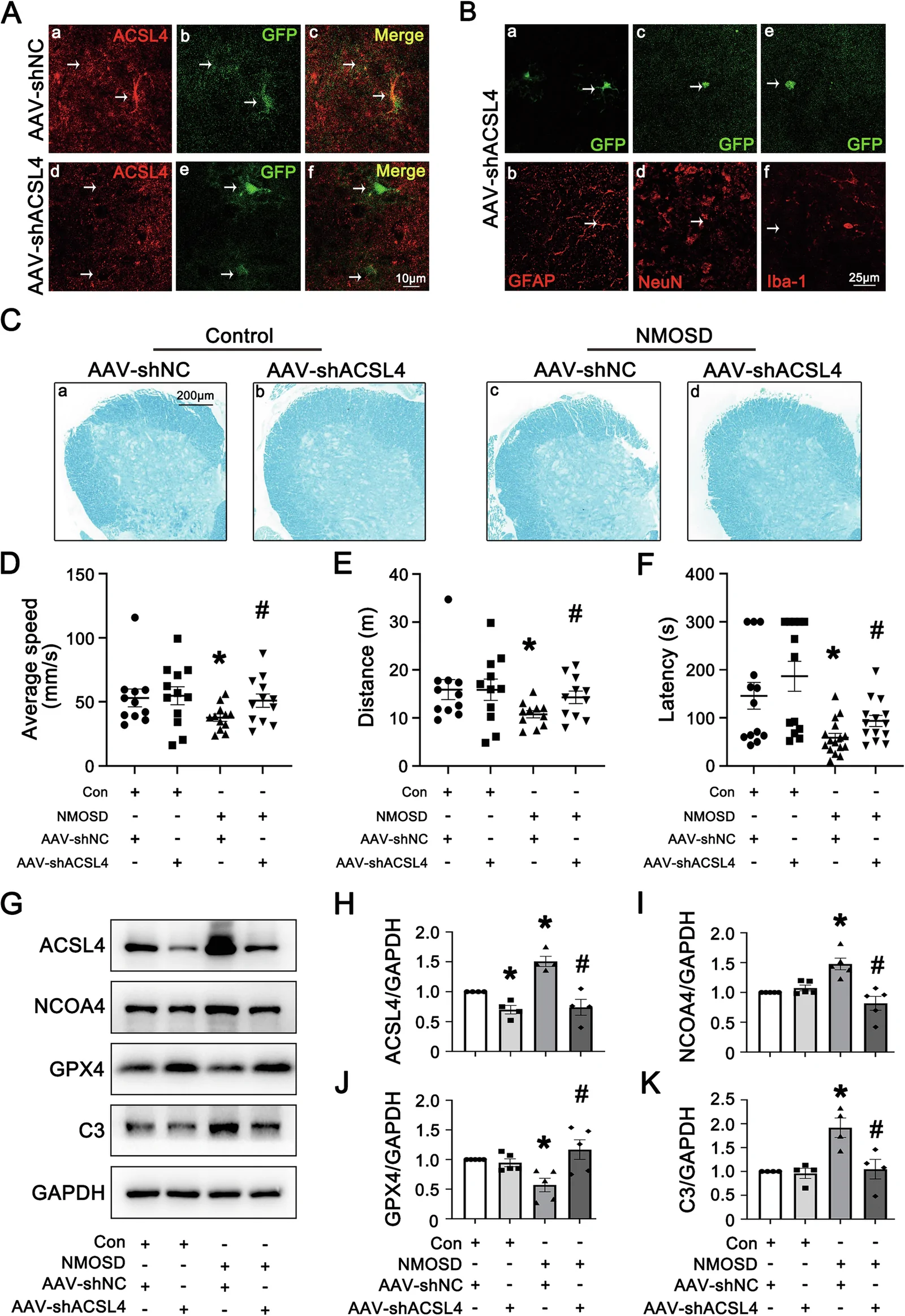
Astrocytic ACSL4 silencing inhibited ferroptosis activation and improved the prognosis of NMOSD mice. C57 mice were intrathecally injected with AAV-shNC or AAV-shACSL4 before NMOSD model induction. The behavioral test was conducted on days 4–5, and mice were sacrificed for further analysis. **A** Typical immunofluorescence image confirmed that an AAV-shACSL4-transfected cell (green) exhibited much lower ACSL4 signal (red) (d-f) than an AAV-shNC-transfected cell (a-c). Scale bar: 10 μm. **B** Typical immunofluorescence images showing that AAV-transfected signal (green) colocalized with GFAP (red) (a&b) but not with NeuN (c&d) and Iba-1 (e&f). Scale bar: 25 μm. **C** Typical images showing paraffin sections of spinal cord stained with LFB to detect myelination. Scale bar: 125 μm. **D** Histogram showing the averaged moving speed of mice during the open field test (*n* = 12 mice per group). **E** Histogram showing the total moving distance of mice during the open field test (*n* = 12 mice per group). **F** Histogram showing the maintenance time on the rotating stick during a 5 min trial of rotarod test (*n* ≥ 13 mice per group). **G** Representative immunoblots showing the expressions of ACSL4, NCOA4, GPX4, and C3 in the spinal cord. **H** Histogram showing ACSL4 expression in spinal cord (*n* = 4 mice per group). **I** Histogram showing NCOA4 expression in the spinal cord (*n* = 5 mice per group). **J** Histogram showing GPX4 expression in spinal cord (*n* = 5 mice per group). **K** Histogram showing C3 expression in spinal cord (*n* = 4 mice per group). Data are expressed as a ratio of the value of Control mice injected with AAV-shNC. Each bar represents the mean ± SEM. *p < 0.05 versus Control mice injected with AAV-shNC. #*p* < 0.05 versus NMOSD mice injected with AAV-shNC.

Lastly, we assessed the role of ACSL4 in NMOSD prognosis by targeted AAV-mediated ACSL4 silencing in vivo. The effective suppression of astrocytic ACSL4 via AAV-shACSL4 was first validated by fluorescent immunohistochemistry (Fig. 8A). Immunofluorescence confirmed that both AAV-shNC and AAV-shACSL4 selectively transfected GFAP-positive astrocytes, instead of neurons and microglia in the spinal cord (Fig. 8B). Luxol fast blue (LFB) staining of spinal cord tissue sections was performed to investigate the effect of AAV-mediated astrocytic ACSL4 knockdown on the demyelination lesion in NMOSD mice. While LFB stain intensity was reduced in NMOSD mice (Fig. 8C, c), it was significantly improved in transplanted animals (Fig. 8C, d). Next, several behavioral tests were conducted to evaluate the impact of astrocytic ACSL4 expression on NMOSD motor function. Open field test results demonstrated that ACSL4 knockdown via AAV-shACSL4 injection significantly improved motor function, as evidenced by increased average speed (Fig. 8D) and longer movement distance (Fig. 8E) compared to AAV-shNC-injected NMOSD mice. Furthermore, NMOSD mice injected with AAV-shACSL4 remained on the rotarod for a significantly longer duration (Fig. 8F), further supporting the improvement in motor dysfunction following ACSL4 silencing in vivo. Western blot analysis demonstrated that AAV-shACSL4 reversed the AQP4-IgG-induced molecular changes in NMOSD mice. Specifically, the increase in ACSL4, NCOA4, and C3, as well as the decline in GPX4 observed in NMOSD mice, was effectively counteracted by ACSL4 silencing (Fig. 8G–K). These findings suggest that astrocytic ACSL4 silencing ameliorates the prognosis of NMOSD mice.

## Discussion

This study provides evidence for the critical role of ACSL4-induced astrocytic ferroptosis in driving astrocytic inflammation during NMOSD pathology. Notably, multiple hallmarks of ferroptosis activation, including abnormal mitochondrial morphology, iron overload, lipid peroxidation, increased MDA and LDH levels, and decreased GSH levels, were observed in NMOSD models, along with a rise in proinflammatory reactive astrocytes. The upregulation of ACSL4 and downregulation of GPX4 were consistently observed in passive and active immunization NMOSD mice, as well as in the in vitro NMOSD astrocyte model. Furthermore, ACSL4 silencing in astrocytes effectively blocked ferroptosis activation, inhibited the A1-like astrocytic phenotype, suppressed proinflammatory factor expression, relieved myelination damage, and ultimately improved motor function in NMOSD mice.

Increasing evidence suggests that ferroptosis plays a role in the pathogenesis of multiple neurological disorders, as iron, a redox-active metal, generates free radicals that induce cell damage and death via lipid peroxidation [27]. Although ferroptosis can occur in multiple CNS cell types—including astrocytes [22], microglia/macrophages [28], oligodendrocytes [29], and neurons [30]—under pathological conditions leading to myelin damage [31], astrocytes exhibit a relatively lower iron storage capacity [32] but enhanced iron uptake ability [33] compared to other neurocytes. Consistently, our study highlights the importance of ferroptosis in astrocytes. Pathogenic antibodies from NMOSD patients induce lipid peroxidation, mitochondrial damage, and glutathione depletion, resulting in astrocyte inflammation. Notably, astrocytic dysfunction may contribute to astrogliosis-related neuroinflammation through an amplification loop under pathological conditions [5, 34]. This amplification loop suggests that ferroptosis-induced astrocytic dysfunction not only drives immediate neuroinflammation but may also predispose astrocytes to a sustained reactive state, further exacerbating CNS damage. These observations position ferroptosis not merely as a downstream consequence of NMOSD pathology but as an active driver of disease progression. The interplay between oxidative stress and immune-mediated astrocytic damage suggests a feedforward mechanism where ferroptosis amplifies neuroinflammation, further sensitizing astrocytes to secondary insults. This cycle may contribute to the persistent and relapsing nature of NMOSD, underscoring the need for early intervention strategies targeting ferroptotic pathways.

Mechanistically, ferroptosis execution involves membrane lipid peroxidation [15], leading to plasma membrane rupture and subsequent cell death. Phosphatidylethanolamines (PEs) containing arachidonic acid (AA) are the primary phospholipids undergoing oxidation [17, 27]. ACSL4 is a key enzyme involved in the synthesis, remodeling, and repair of PE and AA in cellular membranes [35]. Moreover, pharmacological or genetic inhibition of ACSL4 has been shown to counteract ferroptosis [36]. This suggests that ACSL4 inhibition may limit lipid peroxidation-driven astrocytic ferroptosis and modulate astrocyte reactivity, thereby reducing their proinflammatory influence on the CNS microenvironment. Consistently, our results indicate that, by alleviating astrocyte-driven neuroinflammation and preserving astrocyte function, ACSL4 silencing enhances neuronal support and improves synaptic integrity, thereby mitigating NMOSD-associated motor deficits.

Indeed, Egr1 has been reported to transcriptionally upregulate ACSL4 and promote CNS ferroptosis [37]. Consistently, our results demonstrated Egr1 transcriptionally upregulates astrocyte ACSL4 expression in NMOSD model. Yu et al. found genetic ablation of Egr1 or inhibition of its transcriptional activity suppresses the activation of astrocytes and decreases proinflammatory cytokine expression [38], indicating that the induction of Egr1 promotes neuroinflammation. Besides, the activation of Egr1 signaling in astrocytes is related to decreased astrocytic viability, oxidative stress, inflammatory response, and excessive glutamate and ATP release via its regulation of target genes in neurodegenerative disease models [39]. In our study, we confirmed that Egr1 silencing reduced ACSL4 expression and inhibited astrocyte ferroptosis. Taken together, ACSL4 upregulation-induced ferroptosis and further astrocyte inflammation may be under the regulation of Egr1. Specifically, the upregulation of *lysophosphatidylcholine acyltransferase 3 (LPCAT3)*, a gene related to lipid peroxidation, was also observed in the NMOSD model. Notably, extensive research has improved the role of the ACSL4/LPCAT3 pathway in ferroptosis induction [40–42]. Further research might be worth exploring the potential upstream/downstream factors of ACSL4 in order to deeply understand the regulatory mechanism and contribution of ACSL4 in neuroinflammation during NMOSD pathology.

Previous research has demonstrated that compounds targeting oxidative stress and proinflammatory factors can enhance motor recovery following spinal cord injury [43]. Consistent with these findings, our results show that blocking ACSL4 upregulation in astrocytes via genetic inhibition (ACSL4 siRNA and AAV) suppressed ferroptosis-related events, mitigated astrogliosis, and improved motor function in NMOSD mice. Additionally, ferroptosis occurs when iron-mediated free radicals induce lipid peroxidation in the absence of a sufficient glutathione-mediated antioxidant defense (xCT-GPX4-GSH pathway) [42]. GPX4 neutralizes toxic peroxides by oxidizing GSH, thereby protecting cellular membranes from lipid peroxidation [27]. Both in vivo and in vitro NMOSD models, AQP4-IgG exposure disrupted the glutathione-mediated antioxidant system in astrocytes, as evidenced by reduced GPX4 and GSH levels. Notably, these effects were reversed when ACSL4 was genetically inhibited, suggesting a regulatory link between ACSL4 and ferroptosis-associated oxidative damage. ACSL4 and GPX4 are well-established ferroptosis biomarkers [27, 36, 44], yet the potential interactions between ACSL4 and GPX4 in ferroptosis regulation remain largely unexplored. Recent studies indicate that natural flavonoids suppress ferroptosis via the GPX4/ACSL4/ACSL3 axis [45], prompting further investigation into the relationship between ACSL4 and GPX4 in ferroptosis regulation.

In conclusion, these findings raise the possibility that endogenous or pharmacological ACSL4 modulators could fine-tune ferroptotic susceptibility in astrocytes, potentially offering new therapeutic avenues for NMOSD and other neuroinflammatory conditions.

## Materials and methods

### Materials

ACSL4 antibody (#ab155282), SLC7A11 antibody (#ab307601), NCOA4 antibody (#ab86707), GPX4 antibody (#ab125066), and Mounting Medium with DAPI (#ab104139) were purchased from Abcam (Cambridge, UK). COX2 antibody (#12282S) and FTH1 antibody (#4393) were obtained from Cell Signaling Technology (Massachusetts, USA). AQP4 antibody (#PA5-53234), GFAP antibody (#MA5-12023), BCA Protein Assay Kit (#A53225), Opti-MEM™ Reduced Serum Medium (#31985070), fetal bovine serum (FBS, #A3161001C), Image-iT® Lipid Peroxidation Kit (#C10445), Melon™ Gel IgG Spin Purification Kit (#45206), Opti-MEM® Reduced Serum Medium (#31985088), Lipofectamine™ RNAiMAX Transfection Reagent (#13778150), and Lipofectamine™ 3000 Transfection Kit (#L3000001) were obtained from Thermo Fisher Scientific (Massachusetts, USA). Normal Human IgG Control (#1-001-A) was obtained from R&D Systems (Minneapolis, USA). C3/C3b/C3c antibody (#21337-1-AP), S100A10 antibody (#11250-1-AP), β-tubulin antibody (66240-1-Ig), and GAPDH antibody (#60004-1-Ig) were obtained from Proteintech (Wuhan, Hubei, China). Cell Counting Kit-8 (CCK-8, #FD3788) and FerroOrange (#F374) were obtained from Dojindo Molecular Technologies (Tokyo, Japan). Calcein-AM (#C2012), Lipid Peroxidation Malondialdehyde (MDA) Assay Kit (#S0131S), and LDH Cytotoxicity Assay Kit (#C0017) were purchased from Beyotime Biotechnology (Shanghai, China). Reduced Glutathione (GSH) Assay Kit (#A006-2-1) was obtained from Nanjing Jiancheng Bioengineering Institute (Nanjing, Jiangsu, China). SteadyPure Quick RNA Extraction Kit (#AG21023), Evo M-MLV Mix Kit (#AG11728), and SYBR Green Premix Pro Taq HS qPCR Kit (#AG11718) were purchased from Accurate Biology (Hunan, China). RIPA buffer (#FD009), 5× DualColor Protein Loading Buffer (#FD006), FDbio-Dura ECL Kit (#FD8082), Goat anti-mouse IgG (H + L)-HRP (#FDM007), and Goat anti-rabbit IgG (H + L)-HRP (#FDR007) were purchased from FDBIO SCIENCE (Hangzhou, Zhejiang, China). Goat anti-mouse IgG Dylight 549 (#A23310), Goat anti-mouse IgG Dylight 488 (#A23210), Goat anti-rabbit IgG Dylight 549 (#A23320), and Goat anti-rabbit IgG Dylight 488 (#A23220) were obtained from Abbkine Scientific (Wuhan, Hubei, China). Protease Inhibitor Cocktail (#CW2200S) was obtained from CWBIO (Jiangsu, China). Human complement (hC, #S1764) and pentobarbital sodium (#P3761) were purchased from Sigma-Aldrich (Missouri, USA). Complete Freund’s adjuvant (H37Ra, #7027) was purchased from Chondrex (Woodinville, WA, USA). Pertussis toxin (#GC17532) was purchased from GLPBIO (California, USA). The rAAV-GFaABC1D-EGFP-5’miR-30a-shRNA(ACSL4)-3’-miR30a-WPREs and rAAV-GFaABC1D-EGFP-5’miR-30a-shRNA(scramble)-3’-miR30a-WPREs were constructed by BrainVTA Technology (Wuhan, Hubei, China). Three ACSL4-specific small interfering RNAs (siRNAs), a negative control siRNA (siNC), a GPX4 siRNA, an ACSL4 plasmid, and a control vector were designed and obtained from OBiO Technology (Shanghai, China). Liproxstatin-1 (#S7699), RSL3 (#S8155), and Erastin (#S7242) were purchased from Selleck (Texas, USA). Propidium Iodide (PI, #KGA1813) was purchased from KeyGEN BioTECH (Suzhou, Jiangsu, China).

### Mice

C57BL/6 mice (6–8 weeks old) were obtained from Beijing HFK Bioscience Co., Ltd. (Beijing, China) and housed in the experimental animal center of Southern Medical University (Guangzhou, China) under specific pathogen-free conditions. Animals had ad libitum access to water and food and were maintained at a constant environmental temperature (22 ± 2 °C). Following a 7-day adaptation period, all animal experiments were conducted in a blinded manner, with approval and supervision by the Experimental Animal Ethics Committee of Southern Medical University. All procedures were performed in compliance with the NIH Guide for the Care and Use of Laboratory Animals (NIH, revised 1996) and the Animal Research: Reporting In vivo Experiments (ARRIVE) guidelines 3.0. To ensure adequate power to detect the protective effect of ACSL4 silencing, the sample size (number of mice) was chosen based on our prior experience. Mice were randomly assigned using a random number table method. No mice were excluded from the analysis for any reason other than mortality.

### Samples collection and antibody purification

Cerebrospinal fluid (CSF) and serum samples were collected from patients who met the International Panel for NMO Diagnosis 2015 criteria [46]. As previously reported [47, 48], sample collection was conducted with approval from the Research Ethics Committee of Guangzhou First People’s Hospital (Approval Number: K-2023-087-01) and after obtaining full informed consent from patients. CSF and serum samples were centrifuged at 3000 rpm for 5 min, and the supernatants were collected and stored at –80 °C for further analysis.

Patient-derived IgG antibodies were isolated and purified from serum and CSF samples using the Melon™ Gel IgG Spin Purification Kit, following the manufacturer’s instructions. The purified IgG antibodies were then concentrated using a vacuum freeze dryer (LABGENE, Bjarkesvej, Denmark) and redissolved in PBS, ensuring a final concentration of 25 mg/mL.

### Active and passive immunization of NMOSD mouse models

NMOSD mouse models were established as previously described [47, 49]. For the passive immunization NMOSD model, male mice were anesthetized with 1% pentobarbital sodium, followed by intrathecal injection of 10 µL purified patient-derived antibody (25 mg/mL) mixed with 10% human complement (hC) at the L5-L6 spinal cord segments using an insulin syringe for three consecutive days. The tail flick reflex was monitored during injection to confirm that the needle had successfully reached the subdural cavity. Animals were sacrificed on Day 5 after injection, and spinal cord tissues were collected for further analysis.

For the active immunization NMOSD model, 200 μg AQP4 p201-220 peptides (1 mg/mL), emulsified in complete Freund’s adjuvant (CFA, 8 mg/mL) supplemented with Mycobacterium tuberculosis H37Ra, were subcutaneously injected into the back of 8-week-old mice. Control group animals received the same emulsion mixture without the AQP4 peptide. Additionally, 200 ng pertussis toxin was intraperitoneally injected on day 0 and day 2.

### Primary astrocyte culture and treatment

Primary astrocytes were isolated from the cerebral cortex of P1-P3 neonatal mice as previously described [47]. Cells were cultured in DMEM supplemented with 10% FBS at 37 °C in 5% CO₂ for 7 days. To remove microglia, the culture flasks were subjected to shaking at 200 rpm for 2–4 h at 37 °C. Purified astrocytes were then treated with a mixed solution containing AQP4-IgG purified from NMOSD patients or Normal Human IgG Control (CON-IgG) (40 μg/mL) and 5% hC for 24 h to establish the in vitro NMOSD model.

### Single-nucleus RNA sequencing data analysis

Single-nucleus RNA sequencing (snRNA-seq) was performed as previously described [47, 50] using the Chromium Next GEM Single Cell 3’ Reagent Kits v3.1 on the Chromium Controller (10× Genomics). After quality assessment of isolated nuclei, single-nucleus GEMs were formed and barcoded through reverse transcription following the manufacturer’s protocol. Libraries were sequenced on the Illumina NovaSeq 6000 platform with 150 bp paired-end reads at a depth of approximately 0.5 million reads per nucleus. The reads were aligned to the reference using CellRanger (version 6.0.1) to generate the expression matrix of each sample, then subjected to the Seurat (version 4.3.0) platform. High-quality cells were retained with a unique molecular identifier (UMI) < 50,000, captured features’ number < 10,000 and percentage of mitochondria < 10%. After normalization, data were integrated using the SCT method.

Ferroptosis signature score was calculated by gene set variation analysis (GSVA) algorithm with the default parameters. The differentially expressed genes (DEGs) in astrocytes between the Control and NMOSD groups were identified using the Wilcoxon test based on the following criteria: │LogFold Change│ > 0.25, and Bonferroni-adjusted *p*-value < 0.05, with a minimal expression percentage > 0.25 in each group. R package clusterProfiler (version 4.9.0) was used to perform pathway enrichment using Gene Ontology (GO) and KEGG databases.

To identify the potential transcription factors (TFs) regulating ACSl4 activity, we performed Single-Cell Regulatory Network Inference and Clustering (SCENIC) analysis using DEGs between the NMOSD and Control groups by the SCENIC package (version 1.1.0). The correlation between TFs and target genes was calculated using the *runGenie3* function, then the co-expression modules were inferred using the *runSCENIC_1_coexNetwork2modules* function, and regulons were refined through cis-regulatory motif enrichment analysis using the *runSCENIC_2_createRegulons* function. Finally, the activity of each regulon in individual cells was quantified using the *runSCENIC_3_scoreCells* function, where higher scores indicate a greater degree of regulon activation. Afterwards, we extracted the TFs regulating Ascl4 and visualized the regulatory relationships using the igraph package (version 2.0.3).

### Transmission electron microscopy

For electronic microscopic analysis, spinal cord tissues were postfixed in 2% osmium tetroxide with 1.6% potassium ferrocyanide in 0.1 M sodium and sliced into 1 mm^3^ sections. The samples were dehydrated using a graded acetone series and embedded in Eponate 812 medium. Ultrathin sections were double-stained using uranyl acetate. The images were captured using a transmission electron microscope (HT-7700, Hitachi, Tokyo, Japan). Five fields per mice were randomly selected for quantitative analysis.

### Iron concentration measurement

Intracellular Fe²⁺ levels in primary astrocytes were measured using FerroOrange [51], following the manufacturer’s instructions. Briefly, after exposure to AQP4-IgG or CON-IgG, primary astrocytes were incubated in DMEM containing 1 μM FerroOrange for 30 min at 37 °C in the dark. The fluorescent signal of free Fe²⁺ was then immediately captured using a confocal microscope (Nikon ECLIPSE Ti, Chiyoda Ward, Tokyo, Japan) and subsequently quantified using ImageJ software.

### Lipid peroxidation detection

Intracellular lipid peroxidation was assessed using the Image-iT^®^ Lipid Peroxidation Kit [51], following the manufacturer’s instructions. Briefly, Image-iT^®^ Lipid Peroxidation Sensor was added to treated primary astrocytes at a final concentration of 10 μM and incubated for 30 min at 37 °C. After being washed three times with PBS, fluorescence was measured at two excitation/emission wavelengths: 581/591 nm for the reduced dye; 488/510 nm for the oxidized dye. The ratio of emission fluorescence intensities at 590 nm to 510 nm was used as a readout for lipid peroxidation in live astrocytes.

To assess malondialdehyde (MDA) levels, the Lipid Peroxidation MDA Assay Kit was used. MDA detection working solution was added to 0.1 mL of each standard or sample, followed by incubation in boiling water for 15 min. After centrifugation at 1000 × *g* for 10 min at room temperature, 200 μL of supernatant from each sample was transferred to a 96-well plate, and absorbance at 532 nm was measured using a microplate reader. The MDA level was then normalized to protein concentration, which was determined using the bicinchoninic acid (BCA) method.

### Glutathione detection

The Reduced Glutathione (GSH) Assay Kit was used to measure GSH concentration in sample homogenates, following the manufacturer’s instructions. Briefly, 25 μL of Reagent 3 and 100 μL of Reagent 2 were sequentially added to 100 μL of sample supernatant. After thorough mixing, the absorbance at 405 nm was measured using a microplate reader.

### Lactate dehydrogenase (LDH) release assay

The release of lactate dehydrogenase (LDH) was measured using the LDH Cytotoxicity Assay Kit, following the manufacturer’s instructions. Primary astrocytes were treated with patient-derived AQP4-IgG or CON-IgG for 24 h. After treatment, 120 μL of cultured medium was collected and transferred to a 96-well plate, followed by the addition of 60 μL LDH detection working solution to each well.

The 96-well plate was placed on a shaker at room temperature in the dark for 30 min. Absorbance was measured at 490 nm using a microplate reader. Cell toxicity was calculated using the following formula:

$$	 {Cell}\,{toxicity}= \\ 	 \left(\frac{{Absorbance}\,{of}\,{processed}\,{sample}-{Absorbance}\,{of}\,{medium}\,{control}}{{Absorbance}\,{of}\,{cell}\,{maximum}\,{enzyme}\,{activity}-{Absorbance}\,{of}\,{medium}\,{control}}\right)\times 100$$

### Immunofluorescence staining

For in vivo immunofluorescence staining, mice were perfused intracardially with pre-cooled 0.9% sodium chloride, followed by 4% paraformaldehyde fixation. Spinal cord samples underwent post-fixation in 10%, 20%, and 30% sucrose solutions prepared in the same fixative. The tissues were then sectioned into 25 μm-thick axial slices at –22 °C using a Cryostat Microtome (Leica CM1950, Nussloch, Baden-Württemberg, Germany). For in vitro immunofluorescence staining, cells were fixed with 4% paraformaldehyde. Spinal cord sections or cultured cells were then washed with PBS, permeabilized with 0.25% Triton X-100 for 30 min, and blocked with 5% serum for 1 h at room temperature. Samples were then incubated with primary antibodies overnight at 4 °C, followed by Cy3-conjugated or FITC-conjugated secondary IgG antibodies for 2 h at room temperature. Finally, the slices were mounted using Mounting Medium with DAPI for nuclear staining. Fluorescent images were acquired using a confocal microscope (Nikon ECLIPSE Ti, Chiyoda Ward, Tokyo, Japan) and analyzed with ImageJ software.

### Real-time quantitative polymerase chain reaction (RT-PCR)

To assess astrocyte inflammatory activity, total RNA was extracted from primary astrocytes using a SteadyPure Quick RNA Extraction Kit, followed by reverse transcription to cDNA using the Evo M-MLV Mix Kit with gDNA Clean for qPCR. Quantitative PCR analysis was performed using the SYBR Green Premix Pro Taq HS qPCR Kit (Rox Plus) on the QuantStudio™ 5 System (Thermo Fisher) to quantify the expression of interleukin (IL)-6, tumor necrosis factor-α (TNF-α), transforming growth factor-β (TGF-β), interleukin (IL)-1β, zinc-finger E-box-binding homeobox 1 (Zeb1), early growth response 1 (Egr1), and histone deacetylase 2 (Hdac2). Gene expression levels were relatively quantified using the 2ΔΔCT method, with mouse β-actin serving as the endogenous control. The primer sequences used were as follows: IL-6: Forward: 5’-CGGAGAGGAGACTTCACAGAGG-3’, Reverse: 5’-TTCCACGATTTCCCAGAGAACATG-3’;

TNF-α: Forward: 5’-GACGTGGAACTGGCAGAAGAG-3’, Reverse: 5’-TTGGTGGTTTGTGAGTGTGAG-3’; TGF-β: Forward: 5’-ACAATTCCTGGCGTTACCTTGG-3’, Reverse: 5’-GTATTCCGTCTCCTTGGTTCAGC-3’; IL-1β: Forward: 5’-AATCTCGCAGCAGCACATCAAC-3’, Reverse: 5’-AGGTCCACGGGAAAGACACAG-3’;

Zeb1: Forward: 5’-GCTGGCAAGACAACGTGAAG-3’, Reverse: 5’-GCCTCAGGATAAATGACGGC-3’;

Egr1: Forward: 5’-TCGGCTCCTTTCCTCACTCA-3’, Reverse: 5’-CTCATAGGGTTGTTCGCTCGG-3’; Hdac2: Forward: 5’-GGAGGAGGCTACACAATCCG-3’, Reverse: 5’-TCTGGAGTGTTCTGGTTTGTCA-3’; β-actin: Forward: 5’-GTGACGTTGACATCCGTAAAGA-3’, Reverse: 5’-GCCGGACTCATCGTACTCC-3’.

### Western Blot

Total protein from spinal cord tissues and primary astrocytes was extracted using RIPA buffer supplemented with 1% protease inhibitor and 1% phosphatase inhibitor. Protein concentration was determined using the BCA method, and samples were boiled with reducing loading buffer before electrophoresis. Denatured proteins were separated by 8–15% SDS-PAGE and subsequently transferred onto a 0.22 μm PVDF membrane. After blocking with 5% milk for 2 h at room temperature, the membrane was washed with TBST and incubated with primary antibodies overnight at 4 °C. Following additional washes, the membrane was incubated with HRP-conjugated secondary antibodies for 1 h. Immunoreactive bands were visualized using a chemiluminescence imager (5200, Tanon, Shanghai, China) following ECL incubation. The relative optical densities of the protein bands were quantified using ImageJ software.

### Egr1 silence in vitro

To inhibit Egr1 expression in vitro, three small interfering RNA (siRNA) sequences targeting mouse Egr1 and a negative control siRNA (siNC) were designed and generated by OBiO (Shanghai, China). The sequences were as follows: si-Egr1-1 (si-Egr1-756-Mouse): “CUAUCUGUUUCCACAACAATT”, si-Egr1-2 (si-Egr1-1539-Mouse): “CACCUCAAGUGGUCUUUCATT”, si-Egr1-3 (si-Egr1-335-Mouse): “CG﻿AUGGUGGAGACGAGUUATT”, and siNC: “UUCUCCGAACGUGUCACGUTT”.

According to the manufacturer’s instructions, siNC or si-Egr1 was incubated with RNAiMAX reagent for 10 min before being added to primary astrocytes. Culture medium was removed and replaced with fresh medium 24 h after transfection. The silencing efficiency was verified by Western blot, and si-Egr1-1 (si-Egr1-756-Mouse) was selected for subsequent experiments.

### ACSL4 silencing in vitro and in vivo

To inhibit ACSL4 expression in vitro, three small interfering RNA (siRNA) sequences targeting mouse ACSL4 and siNC were designed and generated by OBiO. The sequences were as follows: si-ACSL4-1 (si-ACSL4-649-Mouse): “GUAGAUAUCAAUUGUGUUAAATT”, si-ACSL4-2 (si-ACSL4-985-Mouse): “GCAGAGAUAUCAUGCUUUACCTT”, si-ACSL4-3 (si-ACSL4-1684-Mouse): “GAUGGAUGCUUACAGAUUAUATT”, and siNC: “UUCUCCGAACGUGUCACGUTT”. Primary astrocytes were transfected with siNC or si-ACSL4 as abovementioned, and si-ACSL4-2 (si-ACSL4-985-Mouse) was selected for subsequent experiments.

ACSL4-targeted adeno-associated virus (AAV) was used to specifically induce ACSL4 silencing in astrocytes in vivo. A scramble control AAV vector (AAV-shNC) and rAAV-GFaABC1D-EGFP-5’miR-30a-shRNA(ACSL4)-3’-miR30a-WPREs (AAV-shACSL4) were constructed by BrainVTA Technology. A 2-μL volume containing 11.1 × 10⁹ TU of viral particles was intrathecally injected into the vertebral canal at L5-L6 spinal cord segments. Mice were allowed to recover for 4 weeks after AAV injection to ensure sufficient gene expression before being used in subsequent experiments. The transfection efficiency was verified by Western blot and immunofluorescence staining.

### ACSL4 overexpression in primary astrocytes

Overexpression of ACSL4 in primary astrocytes was achieved through transfection with an ACSL4 plasmid using the Lipofectamine™ 3000 Transfection Kit, following the manufacturer’s instructions. The ACSL4 plasmid or control vector was mixed with Lipofectamine™ 3000 reagent and incubated for 20 min before being added to primary astrocytes. Cells underwent a 24-h transfection process, after which overexpression efficiency was verified by Western blot analysis.

### Calcein/PI staining

Calcein/PI staining was used to visualize astrocyte death following [52]. Briefly, at 24 h after treatment with AQP4-IgG and complement, astrocytes were incubated with Caclein-AM and PI dyes for 20 min. Then, the cells were washed with PBS, carefully avoiding losing dead cells. Finally, the cell death was visualized via a confocal microscope (Nikon ECLIPSE Ti, Chiyoda Ward, Tokyo, Japan) and analyzed with ImageJ software.

### Determination of astrocytic cell viability

The change of astrocytic cell viability was measured by using the CCK-8 assay. Briefly, primary astrocytes were incubated with Erastin or RSL3 for 24 h. Then, cells were incubated with CCK-8 for 2 h. Finally, the OD value at 450 nm was measured with a microplate reader (Synergy HT; Biotech, Winooski, VT, USA).

### Luxol fast blue staining

The paraffin-embedded spinal cord samples were stained with LFB to examine myelination [53]. For LFB staining, the spinal cord sections were baked, dewaxed, and incubated with LFB solution at 60 °C for 16 h. After incubation, the sections were rinsed with 95% ethanol and blued for 5 s using a 0.05% lithium carbonate solution, followed by differentiation with ethanol for 1 min. Finally, the sections were processed with dehydration and mounting before performing microscopic examination. After staining, the sections were sealed and observed under a microscope (ECLIPSE Ci-L, Nikon).

### Behavioral test

Behavioral tests, including the open field test and rotarod test, were conducted on days 4–5 after injection, following previously described methods [46]. For the open field test, mice were placed in an open field arena measuring 40 cm × 40 cm, enclosed by 40 cm-high white walls. Each mouse was allowed to explore the arena for 5 min and their activity was recorded using video tracking software. The open field area was cleaned with a 75% ethanol solution and left to dry before the next test. Total traveled distance and average speed were analyzed to assess motor function in Control and NMOSD mice. For the rotarod test, a rotarod apparatus with an automatic timer was used to assess coordinated movement, balance, and exercise endurance. The rod speed was gradually increased from 4 to 20 rpm during training (day 4) and from 4 to 30 rpm during testing (day 5). The final test lasted 5 min and fall latency was recorded for each mouse.

### Statistical analysis

Statistical analyses were performed using Statistical Package for the Social Sciences (SPSS) Software for Windows, version 25.0 (SPSS, Inc., Chicago, IL, USA). Student’s *t* test (two-sided) was used for comparisons between two groups, while one-way ANOVA (two-sided), followed by Tukey’s post-hoc test, was applied for comparisons involving more than two groups. All variables were expressed as mean ± standard error of the mean (SEM). Differences were considered statistically significant when *p* < 0.05. Scatter plots were generated using GraphPad Prism 8.3.0 (GraphPad Software, La Jolla, CA, USA).

## Supplementary information

Supplementary_Information

Uncropped_WB_blot

## Data Availability

Data was available from the corresponding author upon reasonable request. Data from snRNA-seq have been submitted to the OMIX database (OMIX: OMIX012159).
