# Supplementary material for: ACSL4-mediated astrocyte ferroptosis augments neuroinflammation and exacerbates NMOSD pathology
Source: Cell Death Differ. 2026 Mar 3;33(9):1846–62. doi: 10.1038/s41418-026-01692-y (PMC13545082; doi:10.1038/s41418-026-01692-y)
Supplement: Supplementary file 1 [file 41418_2026_1692_MOESM1_ESM.pdf]

Supplementary Figures

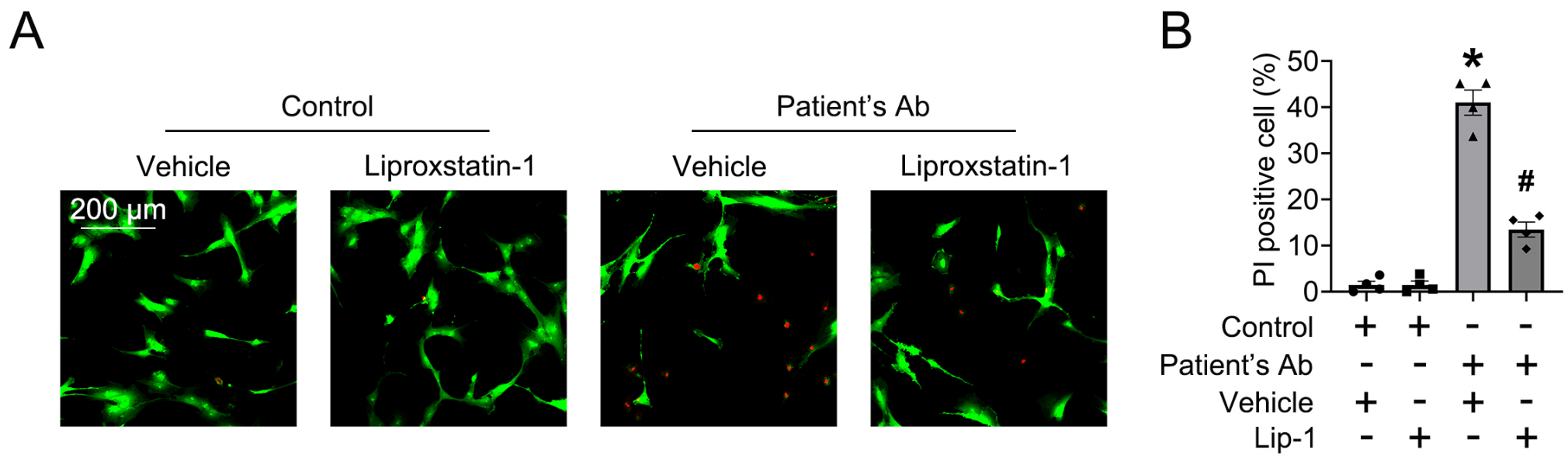

Supplementary Figure 1. Treatment with Liproxstatin-1 ameliorates astrocyte death induced by AQP4-IgG.

(A) Primary astrocytes pretreated with Liproxstatin-1 (Lip-1, 100 nM) were treated with AQP4-IgG or CON-IgG for 24 h. Calcein/Propidium iodide (PI) staining was applied to visualized live cells (Calcein positive, green) and death cells (PI positive, red).

(B) The percentage of astrocyte death was calculated using Image J (n = 4).

Each bar represents the mean ± SEM. *$p < 0.05$ versus Control + Vehicle group. #$p < 0.05$ versus NMOSD + Vehicle group.

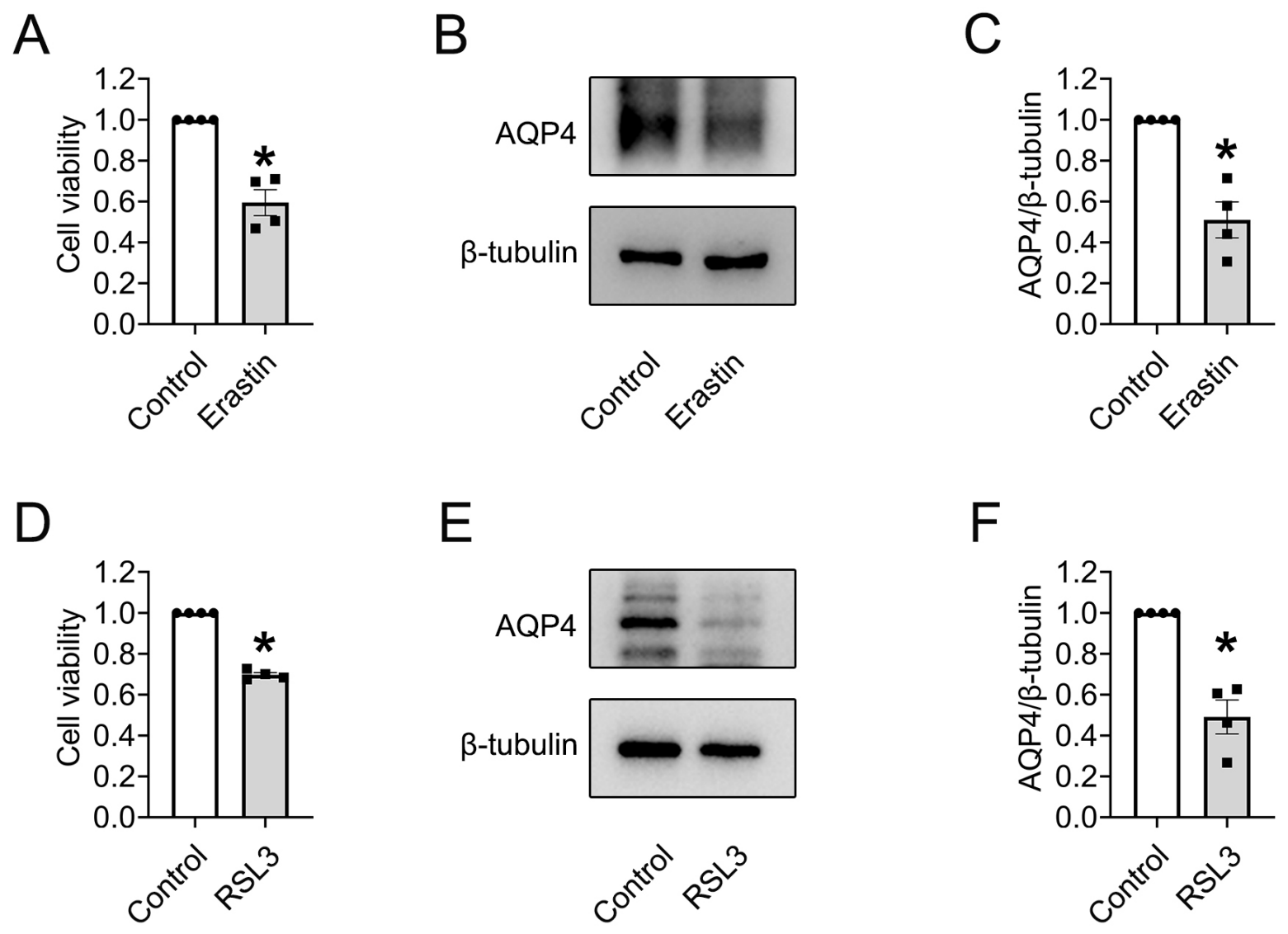

Supplementary Figure 2. Ferroptosis inducers Erastin and RSL3 caused reduction in astrocytic AQP4 expression.

(A) Primary astrocytes were treated with Erastin (40 μM) for 24h, then cell viability was detected by CCK8 assay (n = 4).

(B) Representative immunoblots showing AQP4 expression in primary astrocytes treated with Erastin (40 μM) for 24h.

(C) Histogram showing AQP4 protein level in (B) (n = 4).

(D) Primary astrocytes were treated with RSL3 (0.5 μM) for 24h, then cell viability was detected by CCK8 assay (n = 4).

(E) Representative immunoblots showing AQP4 expression in primary astrocytes treated with RSL3 (0.5 μM) for 24h.

(F) Histogram showing AQP4 protein level in (E) (n = 4).

Each bar represents the mean ± SEM. *$p < 0.05$ versus Control group.

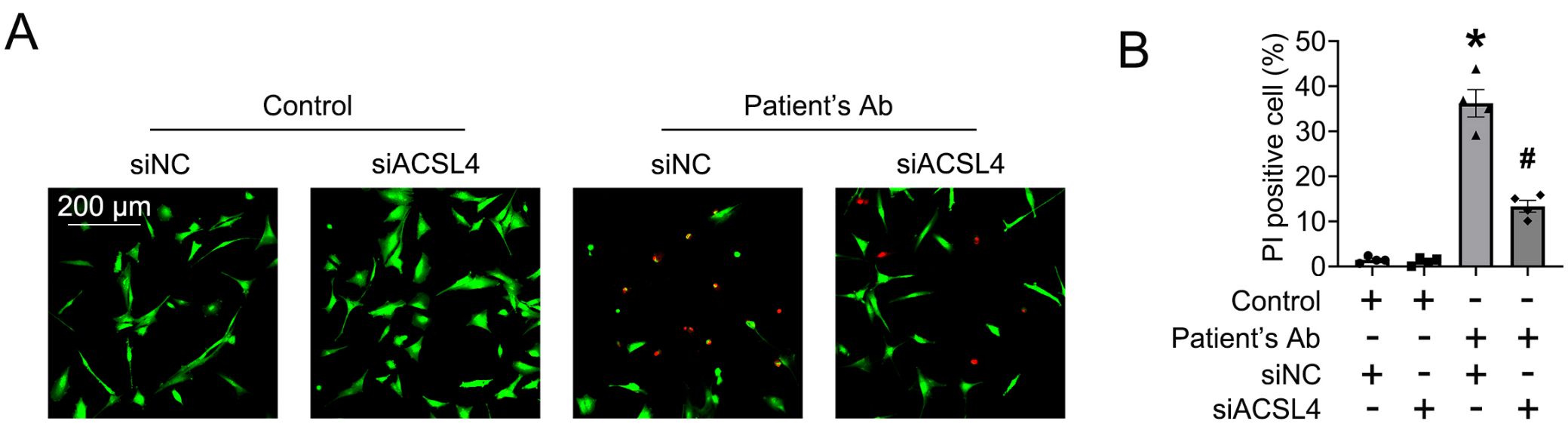

Supplementary Figure 3. Knockdown of ACSL4 attenuates astrocyte death induced by AQP4-IgG.

(A) Primary astrocytes transfected with siNC or siACSL4 were treated with AQP4-IgG or CON-IgG for 24 h. Calcein/PI staining was applied to visualized live cells (Calcein positive, green) and death cells (PI positive, red).

(B) The percentage of astrocyte death was calculated using Image J (n = 4).

Each bar represents the mean ± SEM. *$p < 0.05$ versus Control + siNC group. #$p < 0.05$ versus NMOSD + siNC group.

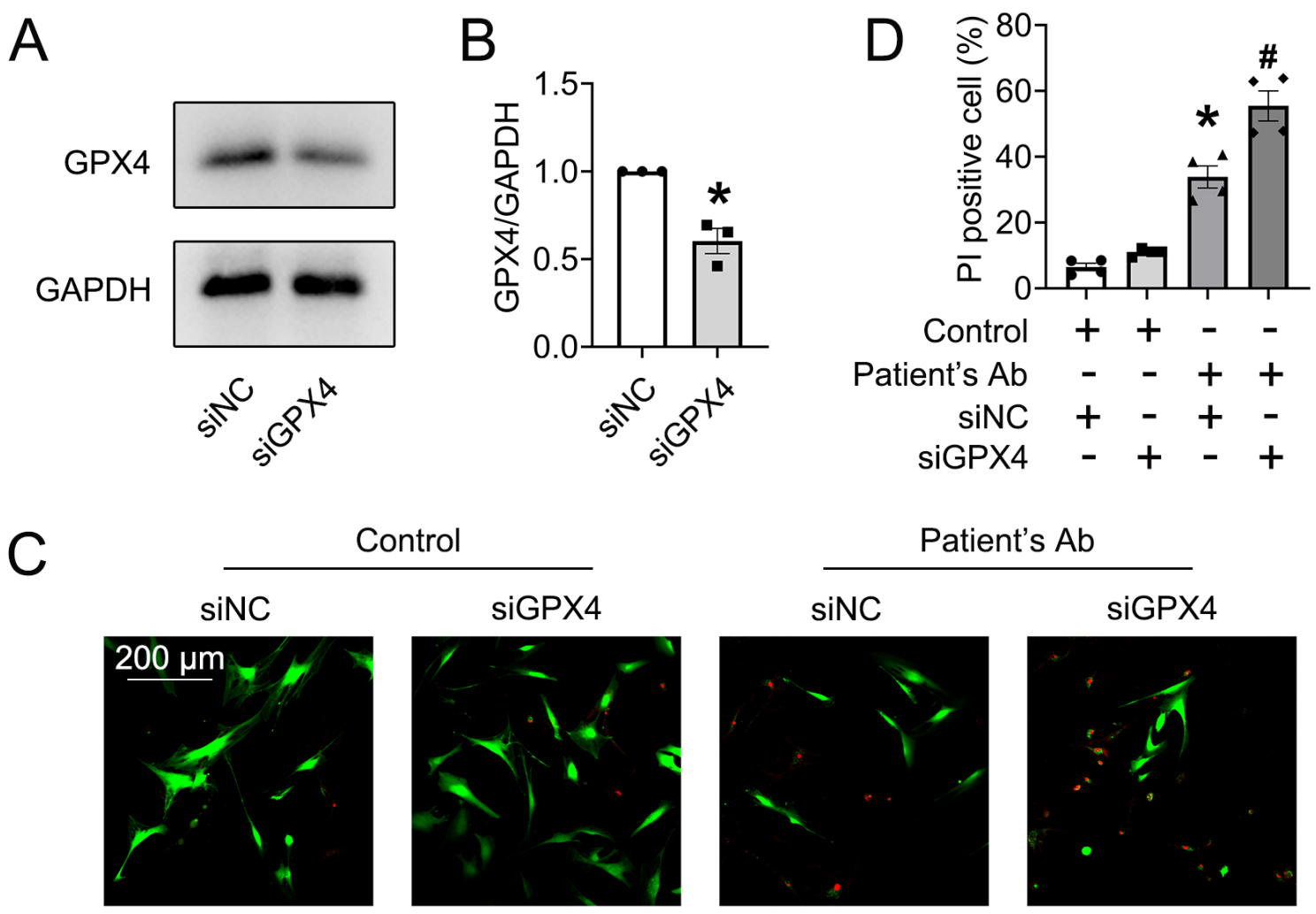

Supplementary Figure 4. Knockdown of GPX4 exacerbates astrocyte death induced by AQP4-IgG.

(A) Representative immunoblots showing GPX4 expressions in primary astrocytes treated with siNC or siGPX4 (Sequence: GCUGCGUGGUGAAGCGCUATT) for 24 h.

(B) Histogram showing GPX4 protein level in primary astrocytes (n = 3). Each bar represents the mean ± SEM. *p<0.05 versus siNC group.

(C) Primary astrocytes transfected with siNC or siGPX4 were treated with AQP4-IgG or CON-IgG for 24 h. Calcein/PI staining was applied to visualized live cells (Calcein positive, green) and death cells (PI positive, red).

(D) The percentage of astrocyte death was calculated using Image J (n = 4). Each bar represents the mean ± SEM. *p < 0.05 versus Control + siNC group. #p < 0.05 versus NMOSD + siNC group.

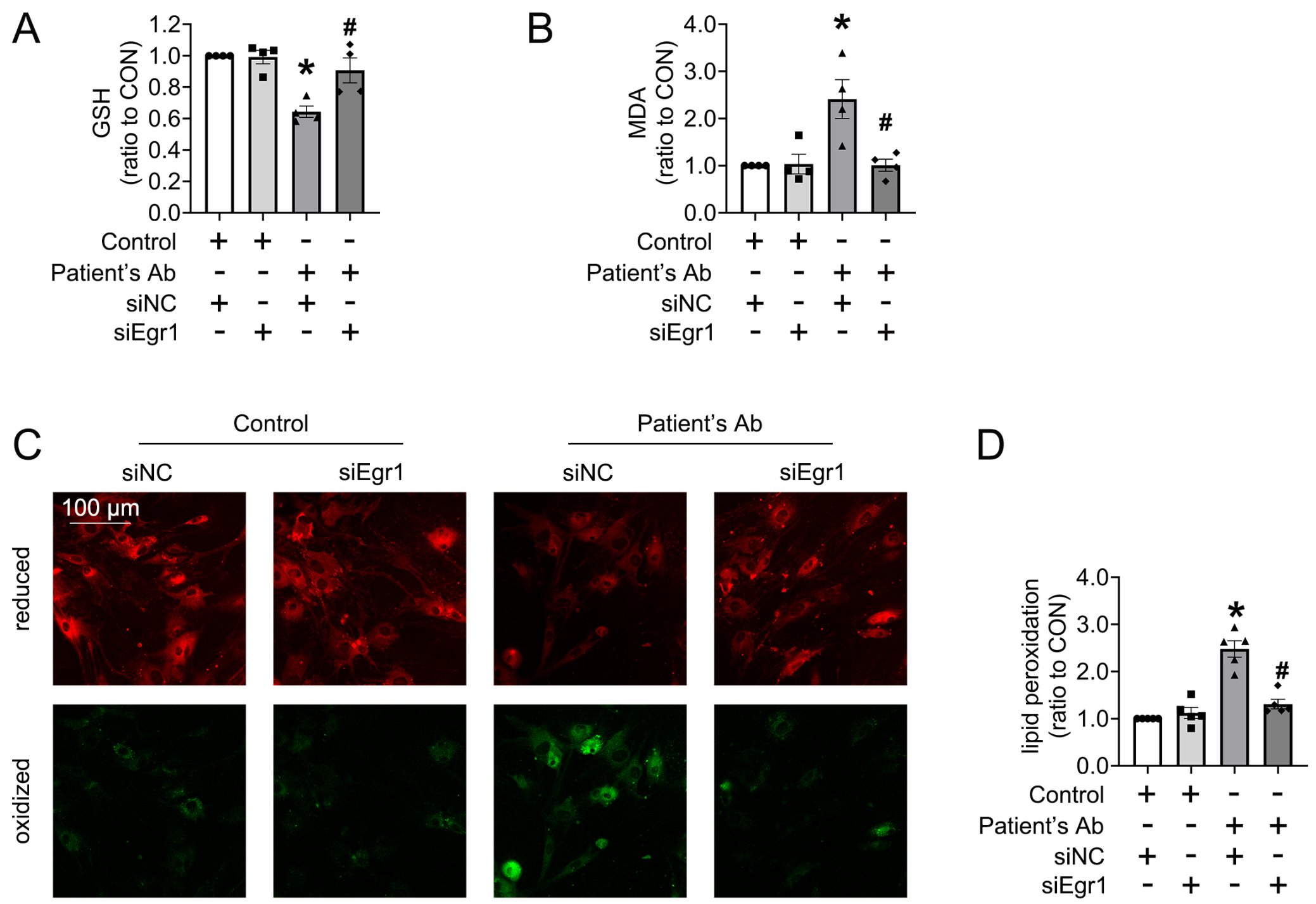

Supplementary Figure 5. Egr1 silencing leads to AQP4-IgG-induced ferroptotic damage.

Primary astrocytes transfected with siNC or siACSL4 were treated with AQP4-IgG or CON-IgG for 12 h.

(A) Histogram showing GSH level in primary astrocytes (n = 4).

(B) Histogram showing MDA level in primary astrocytes (n = 4).

(C) Representative fluorescence images showing the reduced and oxidized signals of lipid peroxidation dye in primary astrocytes.

(D) Histogram showing the level of lipid peroxidation in primary astrocytes (n = 5).

Each bar represents the mean ± SEM. *$p < 0.05$ versus Control + siNC group. #$p < 0.05$ versus NMOSD + siNC group.
