# Supplementary material for: ACSL4-mediated astrocyte ferroptosis augments neuroinflammation and exacerbates NMOSD pathology
Source: Cell Death Differ. 2026 Mar 3;33(9):1846–62. doi: 10.1038/s41418-026-01692-y (PMC13545082; doi:10.1038/s41418-026-01692-y)

Figure 3A

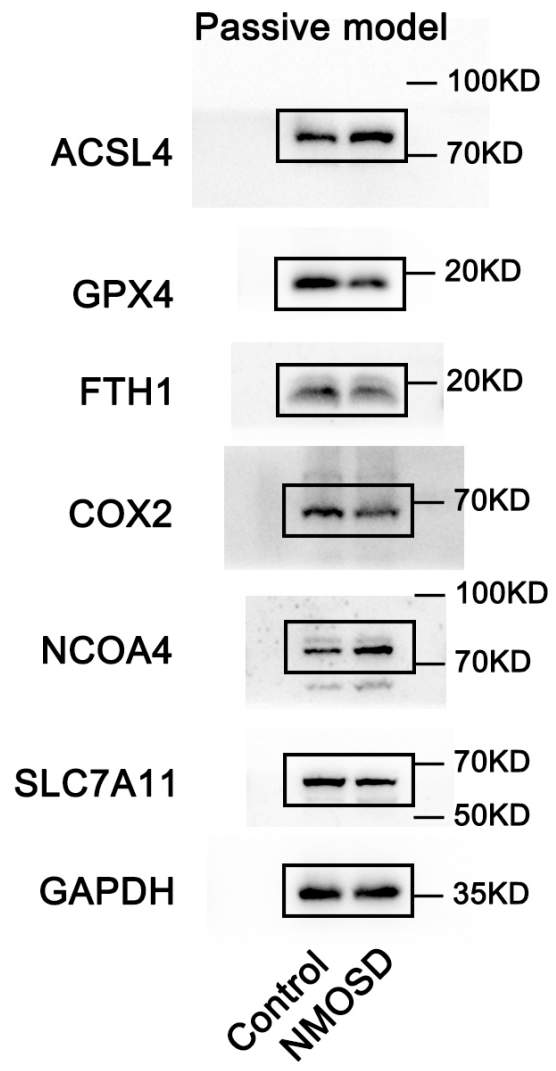

Figure 3K

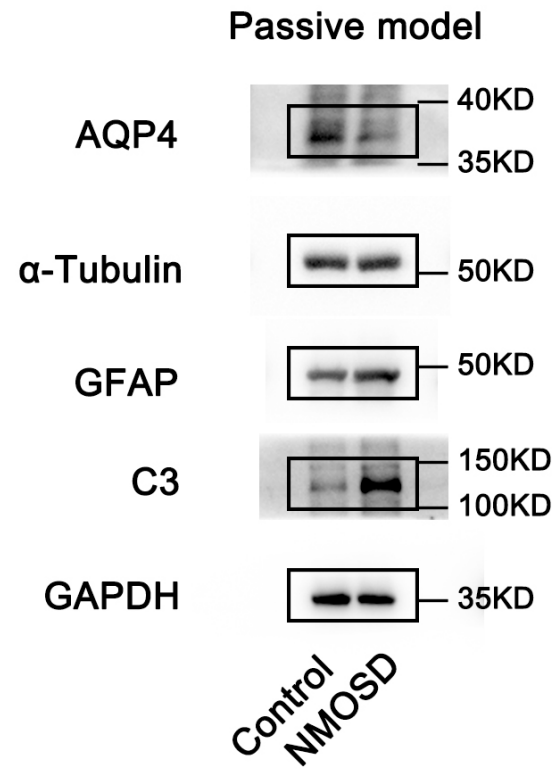

Figure 4A

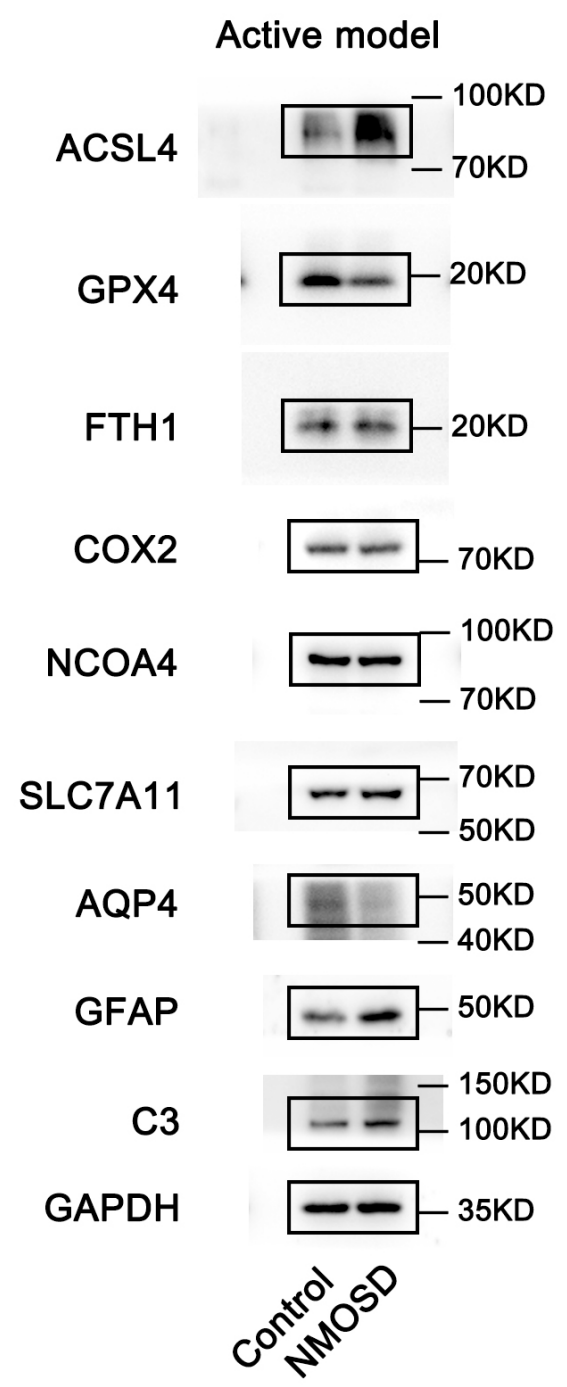

Figure 4K

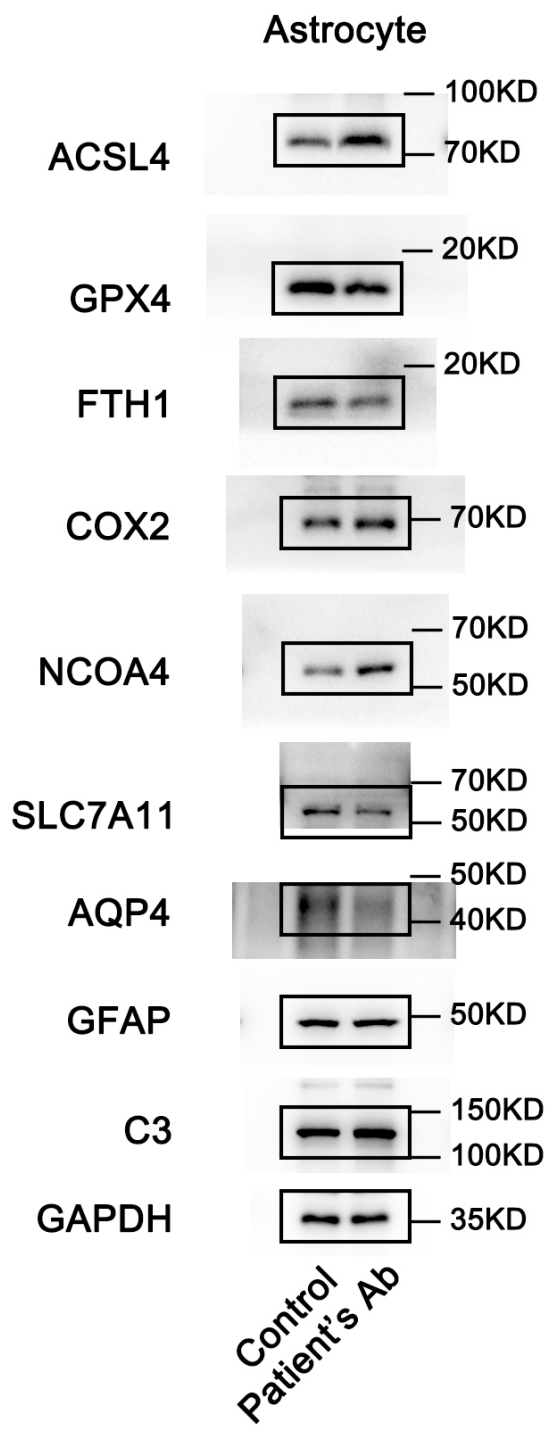

Figure 5F

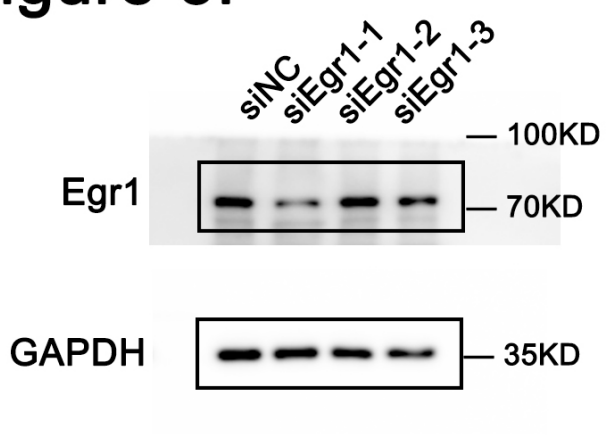

Figure 5G

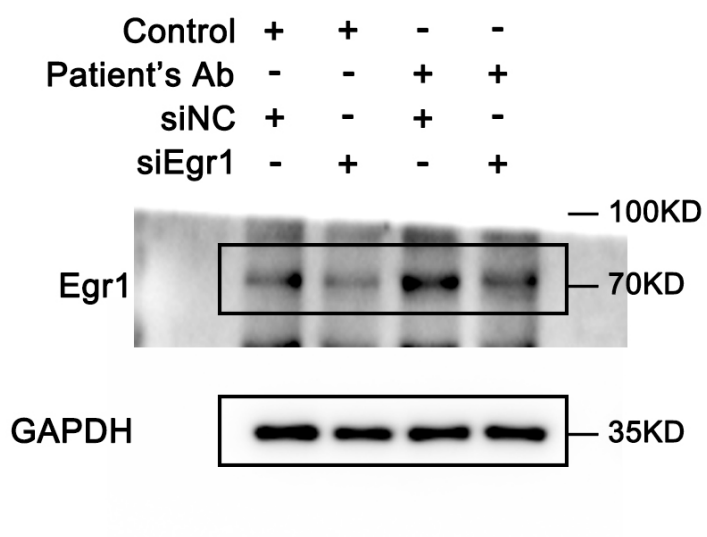

Figure 5H

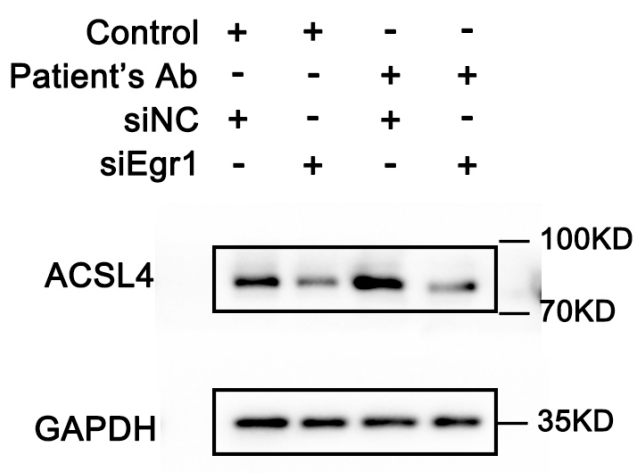

Figure 7I

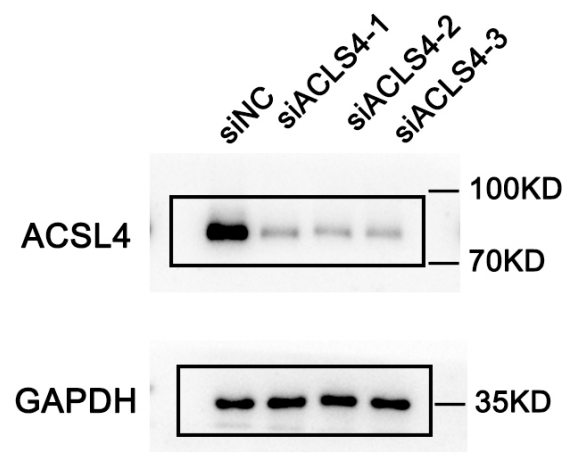

Figure 7J

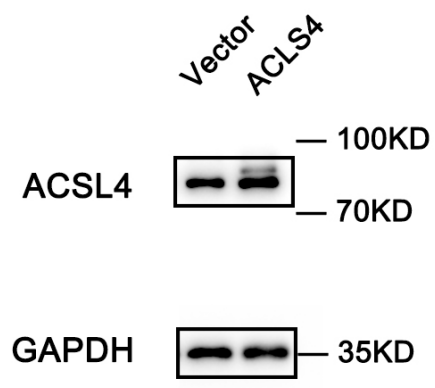

Figure 8G

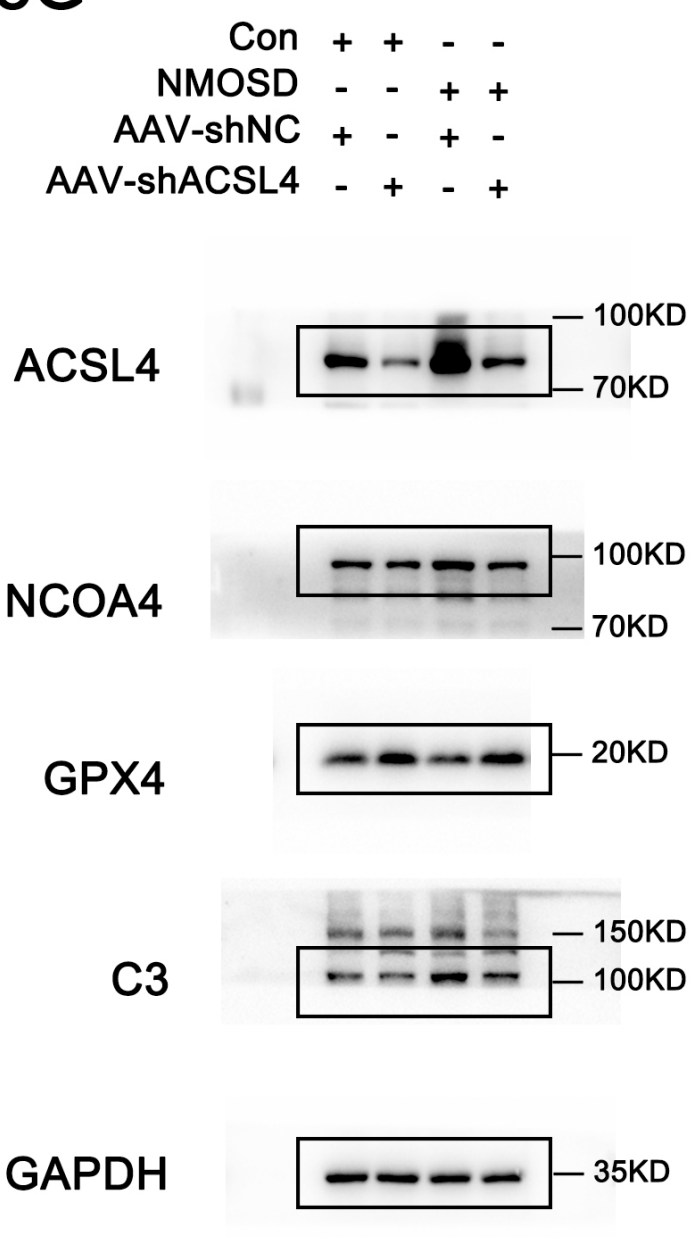

Con + + - -
NMOSD - - + +
AAV-shNC + - + -
AAV-shACSL4 - + - +
ACSL4
100KD
70KD
NCOA4
100KD
70KD
GPX4
20KD
C3
150KD
100KD
GAPDH
35KD

Supplementary Figure 2B

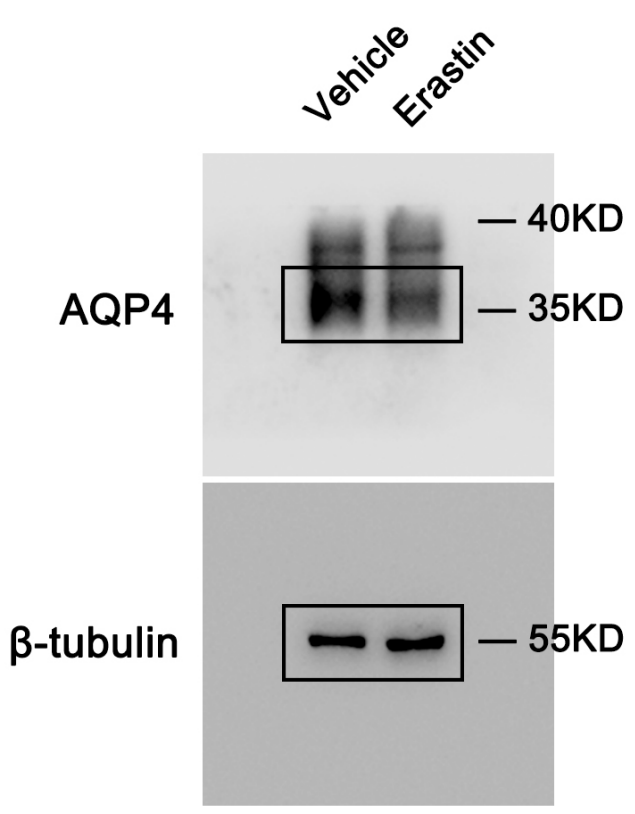

Supplementary Figure 2E

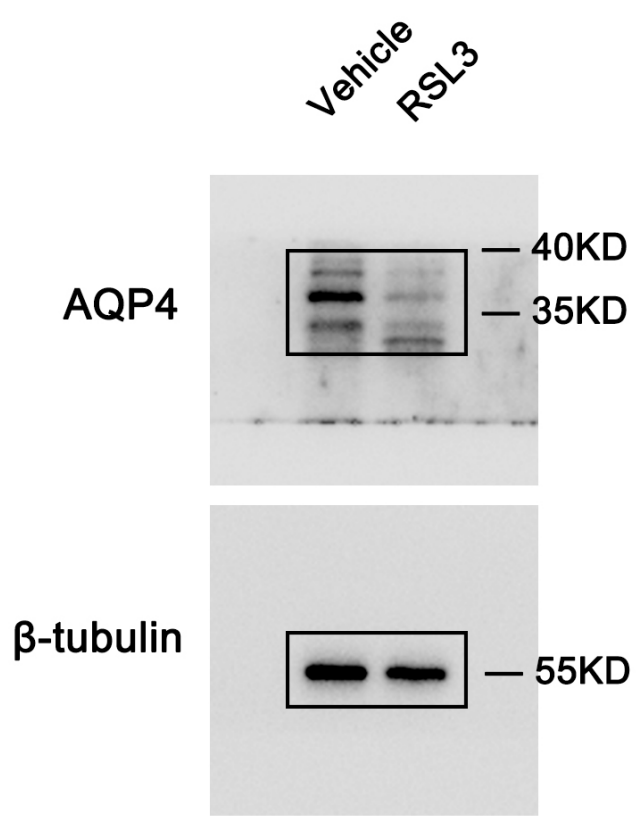

Supplementary Figure 4A

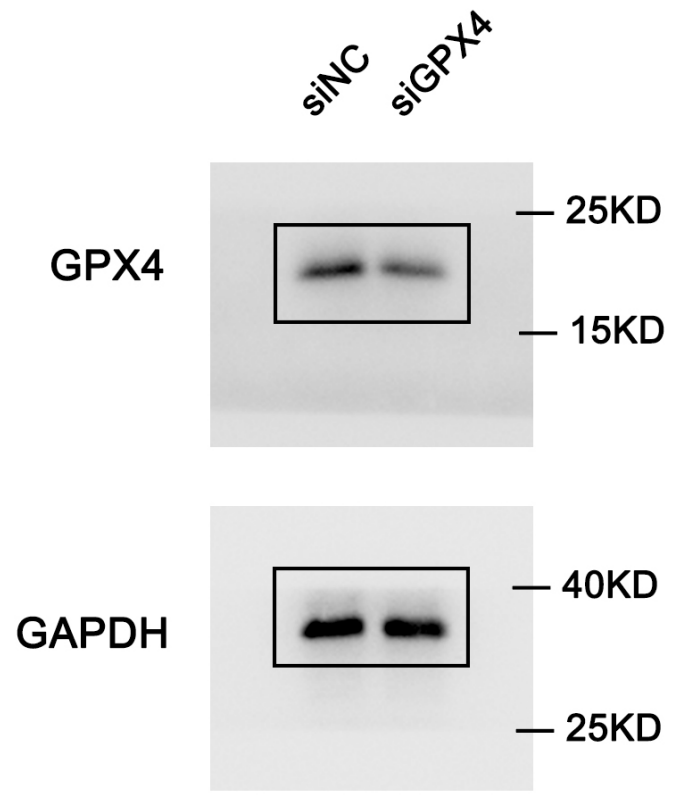

Supplement: Supplementary file 2 [file 41418_2026_1692_MOESM2_ESM.pdf]
